# Poly(ionic liquid)s with Dicationic Pendants as Gas Separation Membranes

**DOI:** 10.3390/membranes12030264

**Published:** 2022-02-25

**Authors:** Sudhir Ravula, Kathryn E. O’Harra, Keith A. Watson, Jason E. Bara

**Affiliations:** 1 Department of Chemical & Biological Engineering, University of Alabama, Tuscaloosa, AL 35487, USA; sravula@ua.edu (S.R.); keoharra@ua.edu (K.E.O.); 2 Department of Chemistry & Biochemistry, University of Alabama, Tuscaloosa, AL 35487, USA; kawatson3@crimson.ua.edu

**Keywords:** poly(ionic liquids), norbornene, ROMP, block copolymers, membranes, gas separations

## Abstract

Poly(norbornene)s and poly(ionic liquid)s are two different classes of attractive materials, which are known for their structural tunability and thermal stabilities, and have been extensively studied as gas separation membranes. The incorporation of ionic liquids (ILs) into the poly(norbornene) through post-polymerization has resulted in unique materials with synergistic properties. However, direct polymerization of norbornene-containing IL monomers as gas separation membranes are limited. To this end, a series of norbornene-containing imidazolium-based mono- and di-cationic ILs (NBM-mIm and NBM-DILs) with different connectivity and spacer lengths were synthesized and characterized spectroscopically. Subsequently, the poly(NBM-mIm) with bistriflimide [Tf_2_N^−^] and poly([NBM-DILs][Tf_2_N]_2_) comprising homo-, random-, and block- (co)polymers were synthesized *via* ring-opening metathesis polymerization using the air-stable Grubbs second-generation catalyst. Block copolymers (BCPs), specifically, [NBM-mIM][Tf_2_N] and [NBM-ImC_n_mIm] [Tf_2_N]_2_ (n = 4 and 6) were synthesized at two different compositions, which generated high molecular weight polymers with decent solubility relative to homo- and random (co)polymers of [NBM-DILs] [Tf_2_N]_2_. The prepared BCPs were efficiently analyzed by a host of analytical tools, including ^1^H-NMR, GPC, and WAXD. The successfully BCPs were cast into thin membranes ranging from 47 to 125 μm and their gas (CO_2_, N_2_, CH_4_, and H_2_) permeations were measured at 20 °C using a time-lag apparatus. These membranes displayed modest CO_2_ permeability in a non-linear fashion with respect to composition and a reverse trend in CO_2_/N_2_ permselectivity was observed, as a usual trade-off behavior between permeability and permselectivity.

## 1. Introduction

Currently, membrane technology is enthusiastically investigated for gas separations due to its energy efficiency relative to well-established technologies utilized for separation of industrial gas streams. This includes H_2_/N_2_ or H_2_/CH_4_ separations, natural gas (CO_2_/H_2_O removal), natural gas sweetening (CO_2_/CH_4_), flue gas stream (CO_2_/N_2_), etc. [1,2,3,4]. Towards this, polymeric membrane-based gas separations are extensively studied in industrial and academic research due to their low energy consumption, cost-effectiveness, reduced process footprint, scalability, and mechanical stability [5]. The polymeric membranes act as a selective layer for efficient separation of gas mixtures, where the gas molecules are absorbed on the upstream surface, transported through the membrane based on their solubility and kinetic diameter, and then desorbed from the membrane through the downstream surface, known as the solution-diffusion (S-D) mechanism [6]. Permeability and selectivity are the key performance metrics of polymeric membranes. Hence, the development of new polymeric membranes with improved ability to selectively transport species of interest (e.g., CO_2_) from other gases is a priority.

Ionic liquids (ILs) are broadly defined as salts with low melting points (<100 °C), generally composed of an organic cation (e.g., imidazolium, ammonium, etc.) and a counter anion (inorganic or organic) [7]. For the past several years, ILs have been significantly expanded in many branches of chemistry due to their unique physicochemical properties including low vapor pressure, excellent thermal stability, and good solvation capability [8,9,10]. Moreover, ILs are considered as designer solvents for task-specific applications. For instance, introducing an amine group to an IL substantially enhances the CO_2_ absorption [11]. Although ILs have shown promising performance in CO_2_/N_2_ and CO_2_/CH_4_ separations, they require substantial volumes for industrial demands [12,13]. In order to compensate, a wide range of ILs have been tested with as “supported ionic liquid membranes” (SILMs) for gas or liquid separations; however, the stability of these SILMs is generally limited to pressure differentials below 1 atm [14]. The best approach to incorporate ILs is chemical doping into the polymeric chains yielding polymerized ionic liquids or poly(ionic liquid)s, poly(IL)s. In general, poly(IL)s, are considered as advanced polyelectrolytes, wherein the ionic moieties are pendant from the backbone of the repeating unit. Combining the salient features of ILs and polymers provides synergistic effects and enhances the intrinsic activity of poly(ILs), which results in a diverse class of functional materials for a wide variety of applications, such as gas separation membranes, fuel cells, catalysts, antimicrobial coatings, etc. [15,16,17,18].

Poly(IL)s have emerged as promising materials for membrane gas separations because of versatile building blocks with a wide variety of functional groups being incorporated in the polymer [19,20,21]. Shen and co-workers were the first to report the enhanced CO_2_ absorption capacity of imidazolium-based poly(IL)s with two different counter anions compared to the analogous bulk ILs [22]. It was indicated that CO_2_ was selectively absorbed compared to other lighter gases (N_2_ or O_2_) without an increase in the weight (due to adsorption) of the membrane. Later, Bara, Noble, and co-workers extensively extended the research area by utilizing the modularity of imidazolium cations tethered to various polymerizable groups (vinyl-, (meth)acrylic-, and stryenyl-) [23,24,25,26,27]. Furthermore, many research groups, including ours, have been focused on developing new poly(IL) materials with significantly enhanced performance by tuning the cation–anion pairs [28,29,30,31,32]. All the above-mentioned glassy poly(IL) membranes resulted in low permeability and diffusivity, but they also showed favorable solubility of CO_2_, which aided in gas separation performance. Additionally, these membranes have high selectivity (CO_2_/N_2_) when compared to SILMs. In spite of extensive research reports that are available on poly(IL) materials for CO_2_ separations, most of the material designs are confined to limited types of polymerizable groups (vinyl-, (meth)acrylic-, and stryenyl-) and common IL cations (imidazolium and pyrrolidinium).

Among the many types of polymeric materials that have been synthesized and investigated for gas separation membranes, substituted poly(norbornene) have emerged as an intriguing materials class [33,34,35,36]. This is because of several advantages, including ease of norbornenyl monomers in gram scale, tunability of structural scaffolds and thermal properties, and amenable to a variety of polymerization mechanisms. However, the substituted poly(norbornene)s have a long history of physical aging and plasticization resulting in the instability of their gas transport properties over time [34]. To minimize the physical aging and improve thermal stability, incorporation of ionic groups in the poly(norbornene) membranes is necessary. In addition, it enhances selectivity because of the advantageous interactions with the polar gases (i.e., CO_2_) within mixtures and enhances the ionic conductivity. There are few examples in the literature that contain poly(NBM-ILs) materials with mono-cationic pendants, obtained either from pre- or post-modification for a variety of applications including self-healing, fuel cells, anti-fouling, etc. [37,38,39,40,41,42,43,44,45]. However, most developments in the field of gas separations came from Gin and co-workers who reported the norbornene-containing hydrophobic alkyl (A), imidazolium cationic group (B) block copolymers (BCP) *via* sequential, living ring-opening metathesis polymerization (ROMP) [39,46]. The authors showed that poly(IL)s-BCP exhibit phase separation and ordered nanostructures that affected the gas separation properties. The CO_2_ permeability of BCPs has greatly increased from 30 to 9300 barrer, when the B content decreased from 100 to 31 wt% but the selectivity (CO_2_/N_2_) decreased drastically from 27 to 9. Similarly, the same group synthesized triblock copolymers of norbornene-containing A, B, and non-charged hydrophilic polyethylene glycol (C) in three order sequences (i.e., ABC, ACB, and CAB) [47]. The reported CO_2_-gas permeabilities values were higher in the ABC (2340 barrer) than the CAB (410 barrer). Unfortunately, the selectivities for the gas pairs CO_2_/N_2_ (10–13), CO_2_/CH_4_ (2.9–4.1), and CO_2_/H_2_ (2.9–4.1) were extremely low. So far, a handful of examples have been reported on poly(NBM-ILs) as gas membranes, and thus it gives us an opportunity to explore different structural designs of poly(NBM-ILs) with enhanced performance by tuning the connectivity and linker groups.

Considering the benefits of poly(IL)s and poly(norbornene)s, herein we explore new designs based on norbornenyl-containing imidazolium ILs. This work reports the synthesis and characterization of norbornenyl-containing imidazolium-based mono- and di-cationic monomers with different alkyl lengths and connectivity. The homo-, random-, and block (co)polymer of norbornene-containing imidazolium dicationic ILs [NBM-DILs] monomers were polymerized *via* ROMP using second generation Grubbs’ catalyst. To our knowledge, there are no such previous reports available on NBM-DILs using ROMP to create gas separation membranes. Further, the purity and other associated properties were investigated and their gas separation behaviors analyzed in terms of pure gas permeabilities and permselectivities. Further, these developed polymers are also suitable for many applications including alkaline fuel cells, catalyst support, solid polymer electrolytes, and ion-exchange membranes.

## 2. Materials and Methods

### 2.1. Materials

1-methylimidazole (99%), 1,4-dibomobutane (98%), 1,6-dibromohexane (97%), dicyclopentadiene (95%), hydroquinone (99%), and dimethylacetamide (DMAc, 99.5%) were purchased from BeanTown Chemical (Hudson, NH, USA). Imidazole (99%) and Grubbs’ catalyst (G2, 97%) were purchased from Sigma-Aldrich (Saint Louis, MO, USA). 1,10-dibromodecane (97%), 1,2-dimethylimidazole (98%), and allyl bromide (99%) were purchased from AlfaAesar (Tewksbury, MA, USA). Lithium bistriflimide (LiTf_2_N) was purchased from 3M (Minneapolis, MN, USA). Ethyl vinyl ether (EVE, >98%) and sodium hydride (NaH, 60 wt% in mineral oil) were purchased from TCI (Portland, OR, USA). Dichloromethane (DCM), dimethylformamide (DMF) acetone, acetonitrile (ACN), diethyl ether (Et_2_O), ethyl acetate (EtOAc), and hexanes were ACS Grade, and purchased from VWR (Atlanta, GA, USA). Unless otherwise noted, all the chemicals were obtained from commercial sources, and used as received.

### 2.2. Characterization

Nuclear magnetic resonance (^1^H and ^13^C NMR) spectra of the newly synthesized monomers and polymers were obtained on a Bruker Avance (500 or Neo500 MHz) instrument using deuterated dimethyl sulfoxide (DMSO-d_6_) as a reference with tetramethylsilane as an internal standard. Fourier transform infrared (FT-IR) spectra of the monomers (liquids) and membranes (solids) were recorded using a Perkin Elmer Spectrum 2 ATR-FTIR spectrometer in the range of 4000−400 cm^−1^ at a resolution of 2 cm^−1^. The number-average molecular weight (M_n_) and polydispersity index (PDI = M_w_/M_n_) of the membranes were determined by gel-permeation chromatography (GPC) analysis (Tosoh Bioscience, Tokyo, Japan) at 40 °C, equipped with a refractive index detector (Bryce, dual flow type) and EcoSEC Elite GPC System Workstation Software. All the measurements were performed using hexafluoroisopropanol (HFIP) as a mobile phase with 0.1% terabutylammonium triflate salts. The eluent flow rate was set to 0.1 mLmin^−1^, using poly(methyl methacrylate) as the standard. The wide-angle X-ray diffraction (WAXD) patterns of membranes were recorded using a Bruker D8 Discover diffractometer by employing a scanning rate of 4° min^−1^ in a 2θ range from 5° to 70° with a Co K_α1_ X-ray source (λ = 0.17886 nm). The interchain distance (*d*-spacing) values were calculated using Bragg’s law (d = λ/2sinθ) and Diffrac-EVA software (Bruker, Billerica, MA, USA). The cross-sections of membranes were captured on a Thermo Scientific Apreo FE-SEM at a magnification between 250X and 500X. The densities of membranes were measured using a Mettler Toledo Density Kit (Product MS-DNY-54), paired with a Mettler Toledo analytical balance. All the measurements were performed in triplicate.

### 2.3. Synthesis

#### 2.3.1. Intermediates 3-(4-Bromo(iodo)alkyl)-1-methyl-1H-imidazol-3-ium Bromide (Iodide) Salts

##### Synthesis of 3-(4-Bromobutyl)-1-methyl-1H-imidazol-3-ium Bromide [C_4_BrmIm][Br]

In a 250 mL round bottom flask (rbf), 1-methylimidazole (15 g, 182.68 mmol) and 1,4-dibromobutane (59.17 g, 274.05 mmol) were mixed in 200 mL of ACN and the reaction mixture was refluxed for 20 h. After cooling to room temperature (RT), the organic solvent was removed under reduced pressure and the viscous light brown liquid was washed with cold EtOAc (3 × 75 mL) and cold Et_2_O (4 × 75 mL). The obtained light brown liquid was dried under reduced pressure followed by vacuum drying at 60 °C for 48 h to obtain the desired product. Yield (48 g, 88%) ^1^H NMR (500 MHz, DMSO-*d*_6_) *δ* 9.30 (s, 1H), 7.85 (s, 1H), 7.77 (s, 1H), 4.26 (t, *J* = 6.9 Hz, 2H), 3.87 (s, 3H), 3.57 (t, *J* = 6.5 Hz, 2H), 1.99–1.87 (m, 2H), 1.85–1.73 (m, 2H).

##### Synthesis of 3-(4-Bromohexyl)-1-methyl-1H-imidazol-3-ium Bromide [C_6_BrmIm][Br]

In a 250 mL rbf, 1-methylimidazole (3 g, 36.54 mmol) and 1,6-dibromohexane (36 g, 146.15 mmol) were mixed in 150 mL of dry acetone and the reaction was stirred at 45 °C for 48 h. After cooling the mixture to RT, the precipitate was removed by filtration and washed with acetone. The organic solvent was removed *via* rotary evaporation. The obtained crude product was washed with cold ethyl acetate (2 × 50 mL) and diethyl ether (2 × 50 mL). The viscous brown liquid was collected by dissolution in a minimal amount of DCM, and the solvent was then removed under reduced pressure, followed by vacuum drying at 60 °C for 48 h to afford desired product as viscous, dark brown liquid. Yield (9.6 g, 81%). ^1^H NMR (500 MHz, DMSO-*d*_6_) *δ* 9.21 (s, 1H), 7.81 (s, 1H), 7.74 (s, 1H), 4.18 (t, *J* = 7.1 Hz, 2H), 3.86 (s, 3H), 3.53 (t, *J* = 6.6 Hz, 2H), 1.80 (qu, *J* = 6.8, 13.8 Hz, 4H), 1.41 (qu, *J* = 7.3, 14.7 Hz, 2H), 1.27 (qu, *J* = 7.6, 14.8 Hz, 2H).

##### Synthesis of 3-(4-Bromodecyl)-1-methyl-1H-imidazol-3-ium Bromide [C_10_BrmIm][Br]

The synthesis of [C_10_BrmIm][Br] IL followed a similar procedure to that of [C_6_BrmIm][Br]. 1-methylimidazole (2 g, 24.36 mmol) and 1,10-dibromodecane (38 g, 121.78 mmol) mixed in 200 mL of dry acetone and the reaction was stirred at 40 °C for 48 h to afford desired product as viscous, pale-yellow liquid (6.5 g, 70%). ^1^H NMR (500 MHz, DMSO-*d*_6_) *δ* 9.16 (s, 1H), 7.79 (t, *J* = 1.6 Hz, 1H), 7.72 (t, *J* = 1.6 Hz, 1H), 4.16 (t, *J* = 7.2 Hz, 2H), 3.86 (s, 3H), 3.52 (t, *J* = 6.7 Hz, 2H), 1.83–1.71 (m, 4H), 1.42–1.32 (m, 2H), 1.31–1.17 (s, 10H).

##### Synthesis of 3-(2-(2-(2-Iodoethoxy)ethoxy)ethoxy)ethyl)-1-methyl-1H-imidazol-3-ium Iodide [((C_2_O)_3_C_2_I)mIm][I]

The oligomer precursor was synthesized according to the reported literature [26,48] and [((C_2_O)_3_C_2_I)mIm][I] IL was synthesized similar to above procedure [C_4_BrmIm]Br. Yield (70%) ^1^H NMR (500 MHz, DMSO-*d*_6_) *δ* 9.07 (s, 1H), 7.74 (s, 1H), 7.71 (s, 1H), 4.35 (t, *J* = 4.6 Hz, 2H), 3.88 (s, 3H), 3.78 (t, *J* = 4.7 Hz, 2H), 3.71–3.62 (m, 2H), 3.60–3.45 (m, 8H), 3.32–3.29 (m, 2H).

#### 2.3.2. Norbornenyl-Containing Imidazolium-Based ILs

##### Synthesis of Norbornylmethylene Bromide (NBM-Br)

The NBM-Br was synthesized according to the literature [37]. In a 250 mL pressure vessel, dicyclopentadiene (30 mL, 224.0 mol), allyl bromide (46 mL, 537.0 mmol), and hydroquinone (150 mg) were added, and sealed properly with Teflon cap and DuPont Kalrez O-ring. The reaction flask was heated at 170 °C for 24 h and the entire setup was covered with glass shield for further protection. After cooling to RT, excess allyl bromide was removed by heating the mixture to 100 °C overnight. The remaining dark viscous liquid was purified by distillation at reduced pressure to afford desired product of a clear, colorless oil as a mixture of *exo* and *endo* isomers. Yield (74%). ^1^H NMR (500 MHz, DMSO-*d*_6_), major isomer: *δ* 6.25 (dd, *J* = 3.1, 5.6 Hz, 1H), 5.98 (dd, *J* = 2.8, 5.6 Hz, 1H), 3.27 (dd, *J* = 7.0, 14.1 Hz, 1H), 3.09 (t, *J* = 9.5 Hz, 1H), 2.92 (s, 1H), 2.85 (s, 1H), 2.51–2.46 (m, 1H), 1.96–1.87 (m, 1H), 1.39 (dd, *J* = 2, 8.2 Hz, 1H), 1.30 (d, *J* = 8.2 Hz, 1H), 0.55 (qd, *J* = 2.9, 4.1, 11.7 Hz, 1H).

##### Synthesis of 1-Norbornylmethylene-Imidazole (NBM-Im)

In a 500 mL rbf, NaH (16.04 g, 668.16 mmol, 60%) in DMF (100 mL) was cooled to 0 °C under Ar atmosphere. After stirring for 15 min at 0 °C, imidazole (27.3 g, 400.89 mmol) dissolved in DMF (100 mL) was added dropwise. The reaction was stirred at RT for another 1 h until the effervescence stopped and the solution color changed from gray to light green. Then, NBM-Br (50 g, 267.26 mmol) in DMF (80 mL) was added drop by drop for 30 min at RT. During the addition process, the color of the reaction mixture changed to a dark wine color and then heated at 120 °C for 24 h. After cooling to RT, the reaction mixture was dissolved with DI water (500 mL) and extracted with DCM (3 × 200 mL). The combined organic layer was washed with DI water (3 × 150 mL), dried over anhydrous MgSO_4_, filtered, and evaporated under reduced pressure. The obtained product was further dried on vacuum at 60 °C for 48 h to afford dark brown oil as a mixture of *exo* and *endo* isomers. Yield (29.5 g, 63%). ^1^H NMR (500 MHz, DMSO-*d*_6_) *δ* 7.61 (s, 1H), 7.17 (s, 1H), 6.88 (s, 1H), 6.25 (dd, *J* = 2.9, 5.6 Hz, 1H), 6.12 (dd, *J* = 2.7, 5.7 Hz, 1H), 3.68 (dd, *J* = 6.9, 13.6 Hz, 1H), 3.53 (dd, *J* = 9.2, 13.6 Hz, 1H), 2.80 (s, 1H), 2.52–2.43 (m, 1H), 1.85–1.72 (m, 1H), 1.33 (dd, *J* = 2.0, 4.0 Hz, 1H), 1.28–1.18 (m, 2H), 0.55 (qd, *J* = 2.6, 4.3, 11.5 Hz, 1H).

##### Synthesis of 3-(4-Bromobutyl)-1-norbornylmethylene-1H-imidazol-3-ium Bromide ([NBM-ImC_4_Br][Br])

In a 250 mL rbf, NBM-Im (13 g, 74.61 mmol) and 1,4-dibromobutane (48.3 g, 223.82 mmol) were dissolved in 100 mL of ACN and heated at 95 °C for 24 h. After cooling to RT, the reaction mixture was evaporated under reduced pressure and then crude viscous mixture was washed with Et_2_O (4 × 75 mL). The product was dissolved in a minimal amount of DCM and the solvent was removed under reduced pressure. Finally, it was vacuum dried at 60 °C for 48 h to afford the desired product as dark brown viscous liquid. Yield (32 g, 90%). ^1^H NMR (500 MHz, DMSO-*d*_6_) *δ* 9.37 (s, 1H), 7.87 (s, 2H), 6.29 (s, 1H), 6.14 (s, 1H), 4.25 (t, *J* = 6.8 Hz, 2H), 3.92 (dd, *J* = 7.0, 13.5 Hz, 1H), 3.78 (dd, *J* = 9.2, 13.2 Hz, 1H), 3.57 (t, *J* = 5.5 Hz, 2H), 2.84 (s, 1H), 2.68–2.57 (m, 1H), 2.03–1.89 (m, 2H), 1.89–1.72 (m, 4H), 1.36 (d, *J* = 7.8 Hz, 1H), 1.33–1.22 (m, 1H), 0.61 (d, *J* = 11.4 Hz, 1H).

##### Synthesis of 1-Norbornylmethylene-3-methyl-1H-imidazol-3-ium Bistriflimide[NBM-mIm][Tf_2_N]

In a 100 mL rbf, 1-methylimidazole (8.95 g, 109.01 mmol) and NBM-Br (14.49 g, 77.90 mmol) were mixed and stirred for 48 h at 60 °C under Ar atmosphere. The crude reaction mixture was washed with excess amount of hexane (3 × 50 mL) and diethyl ether (3 × 50 mL) to remove unreacted starting materials. Finally, the brown viscous reside mixture was dissolved in DCM and LiTf_2_N (27 g, 93.45 mmol) in water (150 mL) was slowly added into the above mixture causing metathesis and stirred for 2 h at RT. The organic layer was washed with water (10 × 200 mL) to remove the LiCl salts and finally dried over anhydrous MgSO_4_, filtered, and evaporated under reduced pressure. The resulting viscous liquid was dried under vacuum at 60 °C for 48 h to afford desired product. Yield (30.7 g, 84%). ^1^H NMR (500 MHz, DMSO-*d*_6_) *δ* 9.13 (s, 1H), 7.79 (s, 1H), 7.72 (s, 1H), 6.29 (s, 1H), 6.12 (s, 1H), 3.94–3.82 (m, 4H), 3.81–3.68 (m, 1H), 2.84 (s, 1H), 2.62–2.53 (m, 1H), 1.92–1.78 (m, 1H), 1.51–1.14 (m, 3H), 0.62 (d, *J* = 11.5 Hz, 1H).

##### Synthesis of 1-Norbornylmethylene-3-(4-(3-methyl-1H-imidazol-3-ium)butyl)-1H-imidazol-3-ium di-bistriflimide [NBM-ImC_4_mIm][Tf_2_N]_2_

[NBM-ImC_4_mIm][Tf_2_N]_2_ was synthesized similar to [NBM-mIm][Tf_2_N] with [NBM-Im] and [C_4_BrmIm]Br in ACN. Yield (29.50 g, 95%) after two steps. ^1^H NMR (500 MHz, DMSO-*d*_6_) *δ* 9.19 (s, 1H), 9.08 (s, 1H), 7.84 (s, 1H), 7.85–7.82 (m, 1H), 7.81–7.77 (m, 1H), 7.75–7.73 (m, 4.20 (t, *J* = 6.5 Hz, 4H), 3.90 (dd, *J* = 7.0, 13.5 Hz, 1H), 3.85 (s, 3H), 3.76 (dd, *J* = 9.1, 13.5 Hz, 1H), 2.85 (s, 1H), 2.67–2.53 (m, 2H), 1.94–1.69 (m, 5H), 1.37 (d, *J* = 8.1 Hz, 1H), 1.25 (d, *J* = 8.3 Hz, 1H), (qd, *J* = 2.0, 3.7, 11.7 Hz, 1H). ^13^C NMR (500 MHz, DMSO-*d*_6_) *δ* 138.82, 137.06, 136.46, 132.08, 124.21, 123.12, 122.94, 122.68, 119.95 (q, *J* = 319.63Hz, 4C), 53.19, 49.43, 48.63, 48.49, 43.94, 42.42, 36.25, 29.75, 26.50.2J.

##### Synthesis of 1-Norbornylmethylene-3-(4-(2, 3-dimethyl-1H-imidazol-3-ium)butyl)-1H-imidazol-3-ium di-bistriflimide [NBM-ImC_4_m(2-m)Im][Tf_2_N]_2_

Yield 2.79 g (78%) after two steps. ^1^H NMR (500 MHz, DMSO-*d*_6_) *δ* 9.19 (s, 1H), 7.84 (s, 1H), 7.79 (s, 1H), 7.63 (d, *J* = 3.7 Hz, 2H), 6.31 (s, 1H), 6.12 (s, 1H), 4.21 (t, *J* = 6.5 Hz, 2H), 4.15 (t, *J* = 7.1 Hz, 2H), 3.90 (dd, *J* = 6.9, 13.5 Hz, 1H), 3.75 (s, 3H), 2.86 (s, 1H), 2.64 (s, 1H), 2.58 (s, 3H), 2.60–2.54 (m, 1H) 1.92–1.76 (m, 4H), 1.76–1.65 (m, 2H), 1.38 (d, *J* = 7.2 Hz, 1H), 1.26 (d, *J* = 8.0 Hz, 1H), 0.62 (d, *J* = 11.3 Hz, 1H). ^13^C NMR (500 MHz, DMSO-*d*_6_) *δ* 144.81, 138.79, 136.41, 132.05, 123.11, 122.91, 122.87, 119.95 (q, *J* = 322.52 Hz, 4C), 53.21, 49.41, 47.19, 43.94, 42.42, 39.70, 39.66, 35.11, 29.72, 26.46, 26.28, 9.57.

##### Synthesis of 1-Norbornylmethylene-3-(6-(3-methyl-1H-imidazol-3-ium)hexyl)-1H-imidazol-3-ium di-bistriflimide [NBM-ImC_6_mIm][Tf_2_N]_2_

Yield (9.69 g, 83%) after two steps. ^1^H NMR (500 MHz, DMSO-*d*_6_) *δ* 9.20 (s, 1H), 9.08 (s, 1H), 7.82 (s, 1H), 7.80 (s, 1H), 7.74 (s, 1H), 7.70 (s, 1H), 6.30 (s, 1H), 6.12 (s, 1H), 4.19–4.10 (m, 4H), 3.90 (dd, *J* = 7.0, 13.5 Hz, 1H), 3.85 (s, 3H), 3.76 (dd, *J* = 9.2, 13.3 Hz, 1H), 2.85 (s, 1H), 2.69–2.53 (m, 2H), 1.95–1.69 (m, 5H), 1.38 (d, *J* = 7.9 Hz, 1H), 1.28 (s, 5H), 0.62 (d, *J* = 11.6 Hz, 1H). ^13^C NMR (500 MHz, DMSO-*d*_6_) *δ* 138.79, 136.94, 136.36, 132.07, 124.10, 123.03, 122.93, 122.69, 119.95 (q, *J* = 323.30 Hz, 4C), 53.17, 49.42, 49.27, 49.14, 43.94, 42.42, 39.68, 36.20, 29.72, 29.61, 29.51, 25.41, 25.37.

##### Synthesis of 1-Norbornylmethylene-3-(10-(3-methyl-1H-imidazol-3-ium)decyl)-1H-imidazol-3-ium di-bistriflimide [NBM-ImC_10_mIm][Tf_2_N]_2_

Yield (5.1 g, 72%) after two steps ^1^H NMR (500 MHz, DMSO-*d*_6_) *δ* 9.22 (s, 1H), 9.09 (s, 1H), 7.81 (d, *J* = 6.6 Hz, 2H), 7.75 (s, 1H), 7.70 (s, 1H), 6.30 (dd, *J* = 3.0, 5.5 Hz, 1H), 6.11 (dd, *J* = 2.6, 5.5 Hz, 1H), 4.14 (dd, *J* = 7.4, 14.7 Hz, 4H), 3.90 (dd, *J* = 7.0, 13.6 Hz, 1H), 3.85 (s, 3H), 3.76 (dd, *J* = 9.1, 13.5 Hz, 1H), 2.85 (s, 1H), 2.66–2.55 (m, 2H), 1.91–1.72 (m, 5H), 1.37 (d, *J* = 8.3 Hz, 1H), 1.33–1.15 (bs, 13H), 0.62 (d, *J* = 5.8 Hz, 1H). ^13^C NMR (500 MHz, DMSO-*d*_6_) *δ* 138.79, 137.05, 136.56, 132.06, 124.19, 123.79, 123.12, 122.93, 119.95 (q, *J* = 321.30 Hz, 4C), 53.20, 49.41, 48.94, 48.63, 48.49, 48.47, 43.94, 42.42, 39.66, 36.22, 30.56, 29.73, 26.58, 26.53.

##### Synthesis of 1-Norbornylmethylene-3-(2-(2-(2-(2-(3-methyl-1H-imidazol-3-ium)ethoxy)ethoxy)ethoxy)ethyl)-1-methyl-1H-imidazol-3-ium di-bistriflimide [NBM-Im((C_2_OC_2_)_2_O)Im][Tf_2_N]_2_

Yield (3.01 g, 83%) after two steps. ^1^H NMR (500 MHz, DMSO-*d*_6_) *δ* 9.16 (s, 1H), 9.05 (s, 1H), 7.82 (s, 1H), 7.76 (s, 1H), 7.70 (d, *J* = 7.8 Hz, 2H), 6.30 (s, 1H), 6.12 (s, 1H), 4.39–4.30 (m, 4H), 3.93 (dd, *J* = 7.0, 13.5 Hz, 1H), 3.86 (s, 3H), 3.82–3.71 (m, 5H), 3.57–3.51 (m, 4H), 3.51–3.45 (m, 4H), 2.85 (s, 1H), 2.67–2.54 (m, 2H), 1.91–1.81 (m, 1H), 1.37 (d, *J* = 7.5 Hz, 1H), 1.26 (d, *J* = 7.9 Hz, 1H), 0.62 (d, *J* = 11.4 Hz, 1H). ^13^C NMR (500 MHz, DMSO-*d*_6_) *δ* 138.78, 137.25, 136.75, 132.04, 123.81, 123.26, 123.12, 122.75, 119.95 (q, *J* = 322.0 Hz, 4C), 69.98 (2), 69.97, 69.94, 68.59, 68.52, 53.11, 49.38, 49.29, 49.19, 43.94, 42.43, 39.74, 36.16, 29.69.

### 2.4. Polymerization

#### 2.4.1. Homopolymerization

All the monomers were dried under vacuum at 60 °C for 48 h prior to the polymerization reaction. In a typical polymerization process, 0.5–2.0 g of monomer was added to a 100 mL round bottom flask and 15–20 mL of dry DCM was added under Ar atmosphere. After 10 min of stirring at RT, Grubbs’ catalyst (G2, 0.005 eq) dissolved in 2 mL of dry DCM was slowly added to the above solution. The progress of the polymerization was monitored by talking aliquots and obtaining ^1^H NMR. spectra For *p*[NBM-mIm][Tf_2_N] polymerization, a viscous liquid was observed at the bottom of the flask after 3 h. After 4 h of stirring, the reaction mixture was quenched with an excess amount of ethyl vinyl ether (EVE) and stirred for 30 min and then precipitated in Et_2_O and stirred for another 30 min. The resultant polymer was isolated and dried under vacuum at 60 °C. For other [NBM-DILs] monomers, insoluble products were observed during the reaction. The reaction was quenched, solids were filtered, precipitated in Et_2_O, and dried under vacuum.

#### 2.4.2. Random Copolymerization

The experimental procedure for random copolymerization is similar to homopolymerization. In general, both the comonomers [NBM-mIm][Tf_2_N] and [NBM-DILs] with appropriate feed ratios were dissolved in dry DCM and stirred for 10 min under argon atmosphere. After 10 min of stirring at RT, G2 catalyst (0.005 eq) dissolved in 2 mL of dry DCM was added slowly to the above solution. After few minutes of stirring, insoluble products were observed during the reaction. The reaction was quenched, solids were filtered, precipitated in Et_2_O, and dried under vacuum.

#### 2.4.3. Block Copolymerization

In the case of block copolymerization, first, the [NBM-mIm][Tf_2_N] monomer was polymerized at room temperature under Ar atmosphere using G2 catalyst (0.005 eq). After 4 h of stirring, the second monomer was added and stirred another 4 h or until completion of polymerization. The reaction mixture was quenched with an excess amount of EVE and stirred for another 30 min followed by precipitation, filtration, and dried under vacuum at 60 °C. The feed ratios used in this study were 1.00:0.11:0.005 and 1.0:0.25:0.005 for [NBM-mIm][Tf_2_N], [NBM-DILs][Tf_2_N]_2_, and G2 catalyst. For convenience, the block copolymers [NBM-mIm][Tf_2_N]_m_-*b*-([NBM-ImC_n_mIm][Tf_2_N]_2_)_p_ (when m = 1 eq then p = 0.11 or 0.25 eq) are denoted as BCP*_y_*-C_n_. Where *y* (= 1 and 2) corresponds to equivalents (0.11 and 0.25, respectively) of [NBM-ImC_n_mIm][Tf_2_N]_2_ and n (= 4 and 6) corresponds to the spacer lengths between the imidazolium cations.

### 2.5. Gas Seperation Measurements

As discussed in our previous works, the pure gas permeation measurements were performed on newly developed homopolymer and block copolymers (BCPs) using high-vacuum time-lag apparatus based on the constant volume−variable pressure method [23,29]. The membranes were “masked” on both sides using an adhesive aluminum tape to confine gas permeation through a fixed membrane area of $\frac{1}{2}$ or $\frac{3}{8}$ in. diameter (A = 0.196 or 0.442 in^2^), known as the aluminum ‘tabbing’ method, which showed no signs of effect on the permeability versus using a full membrane [49]. Ideal (i.e., single gas) permeation measurements were conducted at ambient temperature and the feed pressure was ~2 atm (~30 psia) against initial downstream vacuum (<0.01 psia). Pressures and temperatures were monitored and recorded using the most recent version of LabVIEW software (National Instruments, Austin, TX, USA). Typically, the unit was held under dynamic vacuum (<0.01 psia) for 24 h after each measurement. The permeability coefficient of each membrane was determined from the linear slope of the plot of downstream pressure rise versus time plot (*dp*/*dt*) according to the following equation:

(1) $$P=\frac{273}{76}\times \frac{vl}{ATp_{0}}\times \frac{dp}{dt}$$

where *P* is the permeability expressed in barrer (1 barrer = 10^−10^ cm^3^ (STP) cm cm^−2^ s^−1^ cmHg^−1^); V (cm^3^) is the downstream volume; *l* (cm) is the thickness of the membrane; A (cm^2^) is the effective gas permeation area of the membrane; T (K) is the measurement temperature; _0_ (Torr) is the pressure of the feed gas at the upstream; and *dp*/*dt* is the rate of permeate absolute pressure for steady-state. The ideal permselectivity (α_A/B_) for a pair of gases (A and B) was calculated from the ratio of the individual gas permeability coefficients as, P_A_/P_B_.

## 3. Results and Discussion

### 3.1. Monomer Characterization

**Scheme 1:**
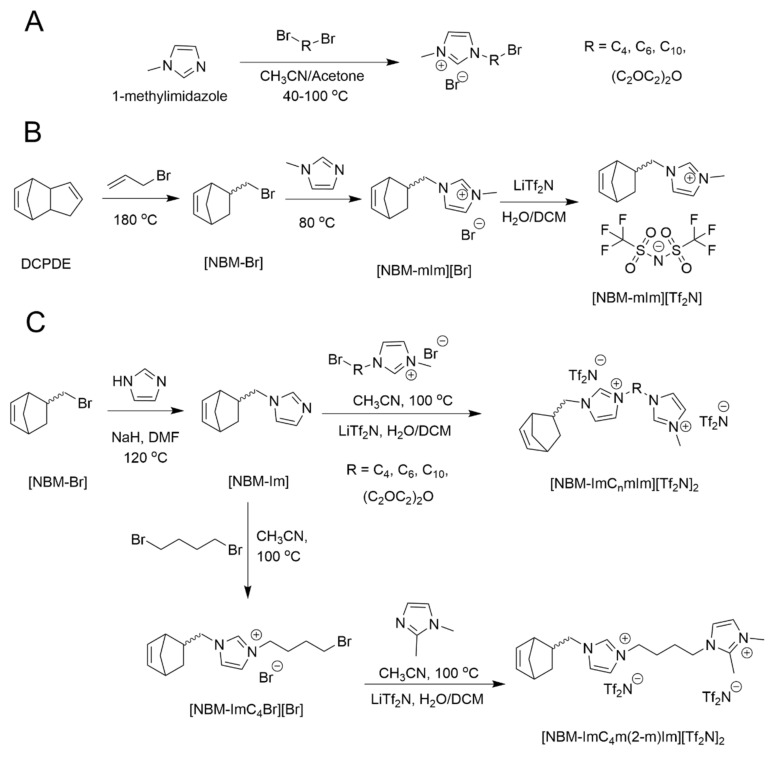
Synthetic routes for intermediate bromoalkyl-containing imidazolium-based bromide salts [C_n_BrmIm]Br (**A**) and norbornenyl-containing imidazolium-based mono- and di-cationic salts, [NBM-mIM][Tf_2_N] monomer (**B**) and [NBM-DIL][Tf_2_N]_2_ monomers (**C**).

A series of new norbornenyl-containing mono- and di-cationic imidazolium ILs with different spacer lengths and connectivity were prepared *via* multi-step organic synthesis. Initially, the intermediates 3-(4-bromoalkyl)-1-methyl-1H-imidazol-3-ium bromide salts were synthesized with 70–88% in yields, see Scheme 1A and experimental section for details. The synthetic routes were shown in Figure S1, and their purity was confirmed by ^1^H NMR, see Figures S2–S5. Further, Scheme 1B shows the synthetic route for norbornenyl-containing imidazolium IL with bistriflimide as a counter anion [NBM-mIm][Tf_2_N], was synthesized in two simple and straightforward reaction steps. Briefly, the first step NBM-Br was synthesized in 70% yield by Diels–Alder reaction of dicyclopentadiene with an excess amount of allyl bromide, resulting in *exo* and *endo* isomers at a 19:81 ratio. The ratio of isomers was confirmed by ^1^H NMR (Figure S6) and match well with the previous literature [37]. Next, a typical Menshutkin reaction between 1-methylimdiazole with NBM-Br, gave imidazolium bromide salts (NBM-mIm][Br]). The bromide salt was purified by washing with cold EtOAc and Et_2_O to remove unreacted starting materials, which resulted in viscous dark brown liquid. Metathesis with a slight excess of LiTf_2_N in aqueous media gave the hydrophobic monomer, [NBM-mIm][Tf_2_N]. 

Similarly, the synthetic routes for norbornenyl-containing imidazolium di-cationic ILs with [Tf_2_N^−^]_2,_ [NBM-DILs][Tf_2_N]_2_ are shown in Scheme 1C. To our knowledge, these ILs have not been reported elsewhere. The NBM-Br was reacted with imidazole through nucleophilic substitution in the presence of sodium hydride, resulting in 1-norbornenemethylene imidazole [NBM-Im] with 60% yield (see Figure S7 for ^1^H NMR). Then, quaternation of NBM-Im with varied bromoalkyl-functionalized imidazolium-based bromide salts in ACN was reacted, and finally metathetic ion exchange with LiTf_2_N as a counter anion, gave [NBM-DILs][Tf_2_N]_2_ with 72–95% yields. Similarly, NBM-Im reacted with excess amount of 1,4-dibromo butane to attain [NBM-ImC_4_Br][Br] IL (see Figure S8 for ^1^H NMR), which can be used as an intermediate for preparing numerous building blocks. In this study, [NBM-ImC_4_m(2-m)Im][Tf_2_N]_2_ IL was prepared from [NBM-ImC_4_Br][Br] and 1,2-dimethylimidazole and exchanged with counter anion. The structures of [NBM-DILs][Tf_2_N]_2_ monomers synthesized in this study were shown in Figure S9.

**Figure 1:**
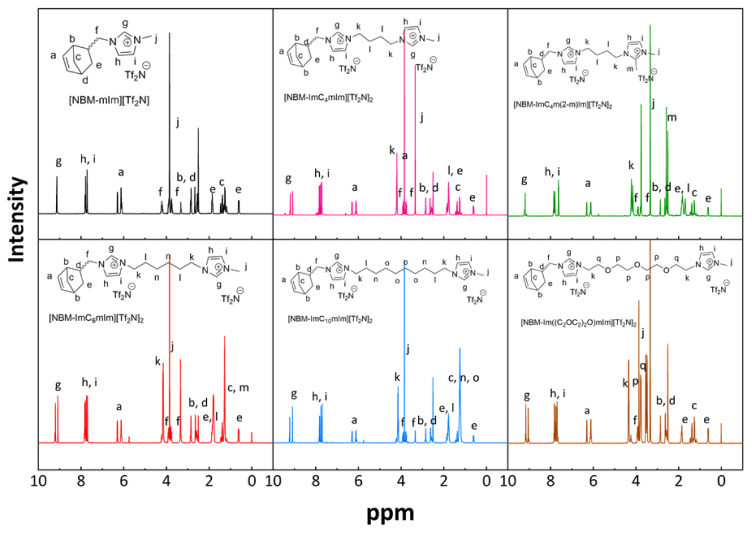
^1^H NMR spectra of [NBM-mIm][Tf_2_N] and [NBM-DILs][Tf_2_N]_2_ in dmso-*d*_6_.

All the imidazolium IL monomers were characterized with NMR (^1^H and ^13^C) spectroscopy. Figure 1 displays the ^1^H-NMR spectra of monomeric compounds of [NBM-mIm][Tf_2_N] and [NBM-DILs][Tf_2_N]_2_. The strong and sharp peaks around *δ* 9.13 pm and *δ* 3.86 ppm corresponds to acidic proton (C(2)-H; H_g_) and substituted *N*-methyl of the imidazolium cation (H_j_), respectively. The characteristic peak for strained NBM moiety was observed at *δ* 6.30–6.07 ppm (H_a_) originates from the olefinic protons and multiplet peaks at *δ* 4.23–3.88 and *δ* 3.78–3.71 ppm corresponds to methylene protons (H_f_) between NBM and Im^+^ cation, respectively. The sharp peak at *δ* 2.55 ppm corresponds to C-2 methyl (H_m_) of pendant Im^+^ cation. The proton peak at *δ* 3.87 ppm is assigned to CH_2_–CH_2_–O– of epoxy groups (H_p_) in [NBM-Im((C_2_OC_2_)_2_O)mIm][Tf_2_N]_2_. Similarly, Figures S10–S14 shows the ^13^C-NMR spectra of [NBM-DILs][Tf_2_N]_2_ monomers. All the aliphatic carbons, epoxy carbon, and alkene carbon were observed around *δ* 53–26, 69–68, and 137–136 ppm, respectively. The quartet carbon peak around *δ* 119.95 ppm was assigned to CF_3_ group of counter-anion, confirming the formation of both cation and anion of [NBM-mIm][Tf_2_N] and [NBM-DILs][Tf_2_N]_2_.

Moreover, the chemical structures of the synthesized monomers were also characterized by FT-IR, as shown in Figure S15. The absorption peaks at 1650 and 722 cm^−1^ in the FT-IR spectrum were associated to C=C (*str*) and C–Br (*str*) of NBM-Br. The peaks around 2869, 2967, and 3063 cm^−1^ correspond to symmetric C-H stretching, and the peaks at around 1330, 1435, and 1220 cm^−1^ correspond to CH_2_ bending of aliphatic carbon chain. On other hand, the new absorption peaks formed at around 3163 cm^−1^ (*ν*_s_ C=CH), 1576 cm^−1^ and 1137 cm^−1^ (*ν*_s_C–N), and 1197 cm^−1^ (*ν*_s_C–C) correspond to Im^+^ cation. Whereas the characteristic peaks of counter anion [Tf_2_N^−^] are observed at around 1344 cm^−1^, 1213 cm^−1^, and 1058 cm^−1^, corresponding to stretching vibrations of SO_2_, CF_3_, and SNS, respectively. All spectral features measured from the liquid samples with different relative intensities, signifying the structural features of as-synthesized imidazolium-based NBM-ILs and confirmed with the literature data [29,37]. Overall, all the NMR (^1^H and ^13^C) signals and FT-IR signals were clearly assigned and matches well with their structures.

### 3.2. Polymer Characterization

**Figure 2:**
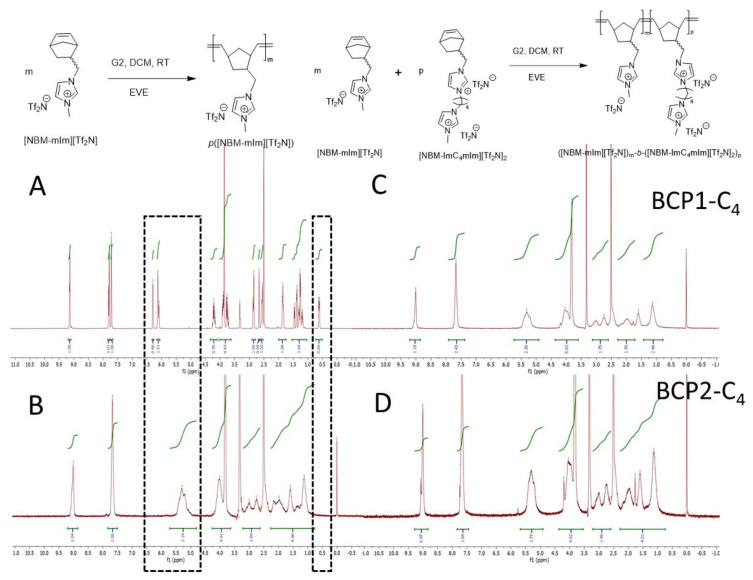
^1^H NMR spectra before (**A**) and after (**B**) homopolymerization of [NBM-mIm][Tf_2_N]. ^1^H NMR spectra of ([NBM-mIm][Tf_2_N]_m_-*b*-([NBM-ImC_4_mIm][Tf_2_N]_2_)_p_) at different equivalents (when m = 1 eq then p = 0.11 or 0.25 eq). (**C**) BCP1-C_4_ and (**D**) BCP2-C_4_ where 1 and 2 corresponds to 0.11 and 0.25 eq of ([NBM-ImC_4_mIm][Tf_2_N]_2_), respectively.

**Scheme 2:**
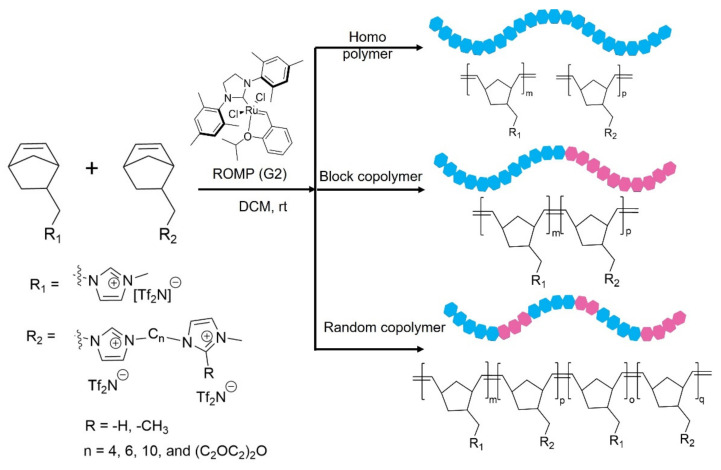
Synthetic routes for homopolymer, block copolymer, and random copolymer of norbornenyl-containing imidazolium-based ILs with different spacer groups and connectivity.

Scheme 2 shows the homo-, random-, and block (co)polymerization of monomers *via* ROMP using the G2 catalyst. The polymerization of [NBM-mIm][Tf_2_N] was carried out in DCM at RT for 4 h under inert conditions and the progress of the reaction was monitored by ^1^H-NMR. In the ^1^H NMR spectra shown in Figure 2A,B, the olefinic peaks at *δ* 6.32–6.05 ppm shifted to *δ* 5.66–4.81 ppm upon ring-opening, and proton peak (H_e_) at *δ* 0.86 ppm shifted upward field, indicating the complete conversion of monomers into polymers was observed. While the peaks from the imidazolium ring were sharp suggesting that Im^+^ is far away from the polymeric backbone and has no effect on the polymerization. The obtained polymer is soluble in most common solvents such as THF, methanol, DMF, DMSO, DMAc, and NMP. The successful ROMP of [NBM-mIm][Tf_2_N] prompted us to prepare the NBM-DILs monomers by modifying the number and connectivity of pendant cation–anion pairs. The homopolymerization of [NBM-DILs][Tf_2_N]_2_ monomers was successful and confirmed with ^1^H-NMR spectra (see Figures S15 and S16). However, these polymers resulted in off-white gum-like solids, which are sparingly or in-soluble in most organic solvents MeOH, ACN, DMSO, NMP, DMF, and DMAc, and observed swelling effects, specifically in DMAc and ACN. To improve the solubility of the *p*([NBM-DILs][Tf_2_N]_2_), different conditions such as reaction time, temperature, reaction solvents, and catalytic amount were investigated and the complete optimization details are shown in Table S1, which only resulted in insoluble polymers during the reaction. Previously, it has been demonstrated that effective polymerization of NBM-ILs with longer alkyl pendant groups were achieved with good solubilities [38,41,42]. From this work, it can be attributed that [NBM-DILs][Tf_2_N]_2_ with varied spacer length between the imidazolium cations and two bulky counter-anions are highly hydrophobic in nature, which might be close to the strained norbornene moiety that makes the entanglement harder and difficult to separate, resulting in high molecular weight polymers with poor solubility. Furthermore, similar results were attained for intermediates ([NBM-Br], [NBM-Im], and [NBM-ImC_4_Br]Br) monomers. The unsuccessful attempts for the linear polymerization of [NBM-DILs][Tf_2_N]_2_ and intermediate monomers *via* ROMPs led us to investigate random and block (co)polymerization. The random copolymerization resulted in insoluble polymers similar to that of [NBM-DILs][Tf_2_N]_2_ homopolymerization, however, the block copolymers are effective and displayed good solubility.

For this study, a four-block copolymerization was prepared between the [NBM-mIm][Tf_2_N] and hydrophobic [NBM-ImC_n_mIm][Tf_2_N]_2_ (n = 4, 6) monomers. The obtained block copolymers [NBM-mIm][Tf_2_N]_m_-*b*-([NBM-ImC_n_mIm][Tf_2_N]_2_)_p_ are composed of different connectivity and equivalents (when m = 1 eq then p = 0.11 or 0.25 eq). For convenience, the block copolymers are denoted as BCP*y*-C_n_ where *y* = 1 and 2 correspond to 0.11 and 0.25 eq of [NBM-ImC_n_mIm][Tf_2_N]_2_, respectively, and n = 4 or 6 corresponds to the spacer length between the imidazolium cations.

First, the [NBM-mIm][Tf_2_N] monomer was stirred for 4 h using G2 catalyst, then the second comonomer with appropriate amounts were added and stirred another 4 h for completion. Beyond these two feed ratios, insoluble polymer was observed during the reaction or sometimes resulted in complete polymerization. The ^1^H NMR was used to investigate the progress of the polymerization and calculated the stoichiometric ratio present in the BCPs. From the ^1^H NMR spectra shown in Figure 2, Figure S17 and Figure S18, BCPs were successfully formed *via* ROMP polymerization. The monomer consumed can be determined from the area of the integrated peaks of vinyl protons δ 5.66–4.81 ppm obtained upon ring-opening and imidazolium acidic protons observed around *δ* 9.13 ppm. The calculated intensities ratios are in accordance with the feed composition used for the block synthesis, implying block copolymerization successfully occurred *via* ROMP. These BCPs showed good solubility in high boiling solvents such as DMAc, NMP, and HFIP. Further, Figure S19 shows no significant difference in the FT-IR spectra after BCPs were observed, suggesting all the peaks were retained. Both ^1^H-NMR and FT-IR clearly suggest that complete block copolymerization was obtained with quantitative yield (>95%).

### 3.3. Membrane Characterization

#### 3.3.1. Gel Permeation Chromatography (GPC)

**Figure 3:**
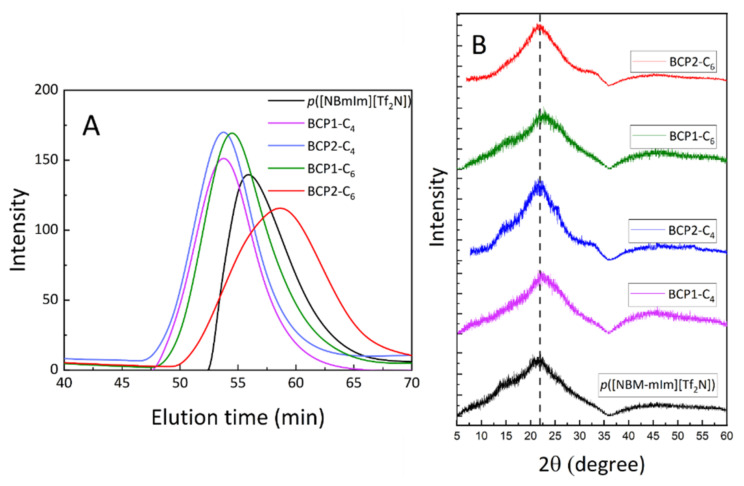
Gel permeation chromatograph (**A**) and wide-angle X-ray diffraction (**B**) plots for the *p*([NBM-mIm][Tf_2_N]) and BCPs membranes.

Figure 3 and Table 1 display the number average molecular weight (M_n_) and polydispersity index (PDI) of the polymers determined with GPC. These polymers showed good solubility in the HFIP solvent, and they took a few hours to attain a homogeneous mixture and the slowest dissolution was observed for BCP2-C_4_, presumably because of high molecular weight. The M_n_ and PDI values for HM (*p*[NBM-mIm][Tf_2_N]) were 32 kDa and 2.01, respectively. The addition of second comonomers to HM, led to a higher M_n_, except for BCP2-C_6_ (M_n_ = 17.4 kDa). The BCPs displayed high M_n_, ranging from 17 to 100 kDa with broad molecular weight distributions (2.02 < M_w_/M_n_ < 3.55), are typical PDI values obtained from the ROMP method [50,51]. The increase in M_n_ is attributed to the successful addition of a second comonomer to the side chains of HM, signifying that the ROMP catalyst remains active until it is quenched with EVE. In addition, no traces of impurity peaks were observed in GPC spectra. The high PDI values for HM and BCPs suggest that the obtained polymers are heterogeneous in nature with a high cross-linking polymer network. Utilizing the M_n_ values and repeat unit MWs (including both cation and anion), the number average degree of polymerization (DP) values was calculated. The DP values for HM, BCP1-C_4_, BCP2-C_4_, BCP1-C_6_, and BCP2-C_6_ are 68, 169, 146, 110, and 25 repeat units, respectively. When the BCP2-C_6_ polymer was redissolved for membrane casting, a fraction of polymer chains was insoluble (possibly cross-linked) and formed a swollen gel. The limited solubility of BCP2-C_6_ polymer might have removed higher molecular weight polymeric chains that could resulted in low M_n_ values and repeating units compared to other BCPs [35,52,53]. Thus, the GPC results confirm the successful addition of [NBM-ImC_n_mIm][Tf_2_N]_2_ (n = 4, 6) monomers through the block polymerization.

#### 3.3.2. Density of Polymers

Based on Archimedes’ principle, the density of the as-synthesized films can be determined [54]. First, the samples were weighed in air and then measured in heptane, a liquid of known density that will not dissolve or swell the ionic polymers. All the measurements were performed at RT using the buoyancy method, and the density was calculated based on the following equation:

(2) $$p_{polymer}=\frac{W_{0}}{W_{0}-W_{l}}\;\times \;p_{liquid}\;$$

where *W*_0_ and *W_l_* are the membrane weights in air and heptane, respectively, and *p_liquid_* is the density of heptane liquid.

Table 1 shows that the average density values of the membranes measured in triplicates are in the range of 1.24–1.53 g cm^−3^. This clearly indicates a linear fashion increase in density value as the hydrophobicity and charged moieties increased. All the four BCPs yielded the highest density values relative to HM suggesting that the molecular architectures in the polymer chains are highly compact giving rise to dense films with reduced free volume, which, in turn, might result in a decrease in gas permeability.

#### 3.3.3. Wide-Angle X-ray Diffraction (WAXD)

To determine the morphology and the ordering patterns between the polymeric chains, WAXD measurements were collected for the HM and BCP derivatives. As depicted in Figure 3, the polymers are lacking crystalline peaks, indicating the amorphous nature of HM and BCPs. These polymers exhibit two broad halos obtained between 5 and 60° (2θ) diffraction angle, with characteristic peaks observed at lower angles. While a broad single-peak distribution was observed for HM with maxima at 21.37° (2θ) with a broad shoulder peak at a lower angle 2θ = ~13.70° was noticed. The shoulder peak disappeared, and the peak sharpness increased in the BCPs with the incorporation of a comonomer, suggesting the greater regularity in the self-assembly and structuring of the matrix. The sharpness of the main halo’s in BCPs were calculated using the full width half maximum, and the values were summarized in Table 1. In addition, the intersegmental (*d*) spacing values of membranes were calculated using Bragg’s law centered between 2θ = 21.37 and 22.63°. As shown in Table 1, the obtained *d*-spacing values are in the range of 4.55–4.75 Å, indicating a dense packing between polymer chains with increased intra- and inter-chain interactions and restricted alkyl chain rotation. However, a clear trend in the *d*-spacing values was observed as the comonomer feed ratio increases. Irrespective of alkyl chain connectivity, the *d*-spacing values for BCP2-C_4_/BCP2-C_6_ increased, indicating that the alkyl groups and imidazolium moieties might be slightly flexible, which might improve the diffusivity values, and, in turn, gas permeability.

#### 3.3.4. Membrane Casting

**Figure 4:**
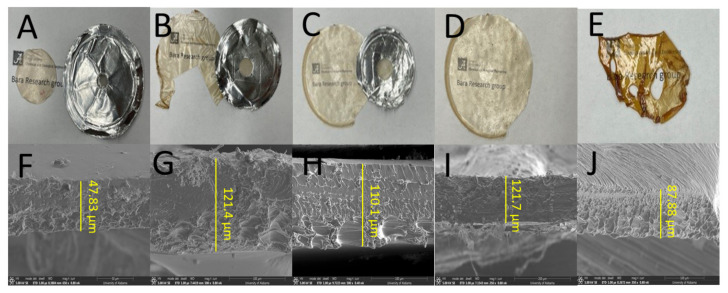
(**A**–**E**) Photographs of the *p*([NBM-mIm][Tf_2_N]) and BCPs membranes, and (**F**–**J**) their corresponding SEM images at a magnification between 250X and 650X. Membrane’s thickness are in the range of 47–125 μm. (**A**,**F**) *p*([NBM-mIm][Tf_2_N]), (**B**,**G**) BCP1-C_4_, (**C**,**H**) BCP2-C_4_, (**D**,**I**) BCP1-C_6_ and (**E**,**J**) BCP2-C_6_.

All the membranes in this study were prepared by solvent casting methods. The HM and BCPs were dissolved in DMAc (1:9 wt/wt) and the mixture was vortexed until a homogeneous solution was attained. The undissolved or dust particles were removed through centrifugation at 3000 rpm for 3 min. It should be noted that undissolved solid polymer was higher for BCP2-C_6_ polymer. Without disturbing the supernatant, the solution was slowly cast on a clean Teflon support followed by slow evaporation of the solvent under vacuum at 40 °C for 48 h. Then, the temperature was slowly raised to 100 °C for a period of 72 h for complete removal of DMAc solvent. After cooling the oven to RT, the membranes can be easily peeled off from the Teflon support and stored at room temperature for further analysis. Figure 4 (upper row) shows the transparent, soft, and flexible membranes obtained from vacuum drying. The softness and flexibility of BCPs decreased with an increase in feed ratio relative to the HM. The cross-sections of the membranes were measured by vernier calipers for initial gas transport studies, and further confirmed with scanning electron microscopy (SEM). Figure 4 (bottom row) shows the SEM images of the membranes, and their cross-sections are in the range of 47–125 μm. In addition, these membranes are flexible and can be fold multiple times (shown in Figure S21) and revert to their original shape without any damage or cracks suggesting the stability of the materials.

## 4. Gas Separations

To study the potential of newly developed *p*([NBM-mIm][Tf_2_N]) (HM) and BCPs for CO_2_ separations, a high-vacuum time lag apparatus based on the constant-volume/variable-pressure method at 2 atm and 20 °C was used. The permeability (*P*), diffusivity (*D*) and solubility (*S*) data for all four gases (N_2_, CO_2_, H_2_, and CH_4_) and permselectivities (CO_2_/N_2_, CO_2_/CH_4_, and CO_2_/H_2_) are summarized in Table 2 and Table S2.

The *P* of HM for CO_2_, N_2_, CH_4_ and H_2_ are 6.33 ± 0.37, 0.29 ± 0.03, 0.31 ± 0.02, and 3.98 ± 0.13 barrer, respectively. Interestingly, the HM displayed a very low *P*_CO_2__ (6.33 ± 0.37) compared to previously reported homopolymer *p*([NBM-C_6_Im][Tf_2_N]) (30 barrer) with longer pendant chain [46]. This is a common trend for light gases in poly(IL)s as the chain length influences the transport properties. The lengthy alkyl side chains create more free volume within the polymer domains due to the inefficient packing of alkyl side groups [23]. Thus, the HM with pendant methyl group exhibits a distinct backbone architecture obtained through the polymerization process, which created compact polymer chains with reduced free volume.

On other hand, the *p*_CO_2__ values of BCPs are in the range of 6–11 barrer and the *P*_N_2__, *P*_CH_4__, and *P*_H_2__ permeabilities values differ from 0.21–0.51, 0.29–0.58, and 3.86–7.69 barrer, respectively. It is understandable that the permeabilities of all four gases are low for BCPs because of the low *P* values demonstrated by the HM. However, BCPs displayed higher *P* values after incorporation of [NBM-ImC_n_mIm][Tf_2_N]_2_ (n = 4, 6) to HM at two different stoichiometric ratios. Among these four BCPs, BCP1-C_4_ and BCP1-C_6_ displayed higher *P*_CO_2__, approximately 1.5 to 2 times higher than the HM. The slight increase in *P* might be due to increased di-cationic pendant groups within the polymer chains; although, they have similar *d*-spacing values and higher polymer density relative to HM. Interestingly, the *P* values decreased for BCP2-C_4_ and BCP2-C_6_ with an increase in feed ratio, leading to strong interchain interactions with high dense packing. However, it should be noted that the obtained *P* values for these BCP membranes are closely matching with the previously reported poly(IL)s data [27].

The gas permeabilities of these gas membranes depend upon the *D* and *S* data. Using the time-lag experiments, the diffusion coefficients were determined from the slope of steady-state flux, and the *S* was calculated from the *P* and *D*. The *S* and *D* values for some BCP membranes could not be determined because of low flux values obtained from the time-lag experiments. Table S2 clearly shows the *D* values for N_2_ and CH_4_ are low compared to CO_2_ and H_2_, which follows the order of their kinetic diameters: H_2_ (2.89 Å) > CO_2_ (3.3 Å) > N_2_ (3.6 Å) > CH_4_ (3.8 Å). Even though *D*_CO_2__ is improved to 5–15 fold for the BCPs, the increase is quite minimal when compared with the previously reported poly(IL) membranes [12,13,23]. This could be due to the presence of cross-linked chains and the increased hydrophobic counter anions that might inhibit the gas molecules to diffuse from the dense films. This is further corroborated with the density and XRD data, where the density and *d*-spacing of BCPs increase with respect to the stoichiometric composition. Overall, the *D* values show a non-linear trend within the BCPs for butyl and hexyl connectivity, signifying that the charged-alkyl pendant groups increased the diffusivity of all gases, which, in turn, enhances the permeability values. Interestingly, the solubilities of BCPs are 4 to 5 times lower than the HM, which is not typical for charged ionic polymers [22,23]. The incorporation of [NBM-ImC_n_mIm][Tf_2_N]_2_ monomers at different feed ratios should improve the *S*_CO_2__ of BCPs more than that of HM because of an increase in the polar imidazolium component. However, the increase in ionic groups in BCPs might be trivial when compared to the hydrophobic content, which might result in the dilution of *S*_CO_2__ [12,23]. This further validates with unaffected *S*_CH_4__ values for BCPs. Based on the *D* and *S* values shown in Table S2, the BCP2-C_4_ and BCP2-C_6_ membranes are more favored for CO_2_/N_2_ separation because of moderate *D*_CO_2__ and *S*_CO_2__ values, and low *D*_N_2__ rate.

**Figure 5:**
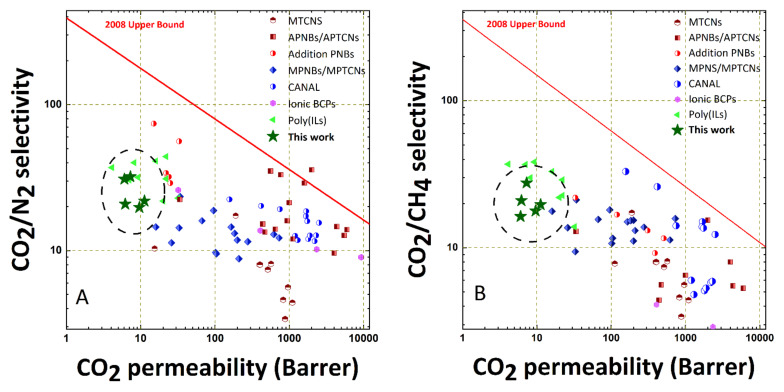
Robeson upper bound plot [55] for comparing the (**A**) CO_2_/N_2_ and (**B**) CO_2_/CH_4_ separation performance of the studied *p*[NBM-mIm][Tf_2_N] and BCPs membranes. Data from the reported literature values poly(IL)s [24,25,27], ionic BCPs [39,46], and norbornene-based polymers [34] were taken for comparison purposes.

The permselectivities (α) of CO_2_/N_2_, CO_2_/CH_4_, and CO_2_/H_2_ values are reported in Table 2 and plotted on the so-called Robeson plots in Figure 5. From Table 2, α_CO_2_/N_2__ values are more favorable than α_CO_2_/CH_4__ and α_CO_2_/H_2__ for membranes prepared in this work because the *P* of CH_4_ is slightly higher than that of N_2_. This trend in permselectivities is consistent with most of all previously reported poly(IL)s membranes [22,23,24]. The α_CO_2_/N_2__ and α_CO_2_/CH_4__ for HM are 20.76 ± 0.41 and 20.91 ± 0.19, respectively, which is comparable to the previously reported *p*([NBM-C_6_Im][Tf_2_N])(α_CO_2_/N_2__ = 27) [46]. Further, modifying the BCPs with butyl- and hexyl-ionic groups at different compositions has significantly improved the α_CO_2_/N_2__ and α_CO_2_/CH_4__ of the BCP membranes. For instance, the α_CO_2_/N_2__ for BCP2-C_4_ and BCP2-C_6_ are 31.27 ± 1.34 and 30.83 ± 0.27, respectively, showed excellent permselectivities among the four BCPs. On contrary, the *P* values for BCP1-C_4_ and BCP1-C_6_ are relatively high but the α_CO_2_/N_2__ remains unchanged relative to HM due to the high diffusivity of N_2_.

The separation performances of studied membranes were shown in the Robeson Plot (Figure 5) and compared with other reported poly(IL)s [27], ionic BCPs [39,47], and norbornene-containing polymers (MTCNs, APNBs/APTCNs, addition PNBs, MPNS/MPTCNs, and CANAL) [34]. The data for norbornene-containing polymers were extracted from the review article, and were synthesized through different polymerization mechanisms. The “upper bound” [55] shows the tradeoff between the *P*_CO_2__ and α(CO_2_/N_2_ and CO_2_/CH_4_) for the reported polymers in Figure 5. For instance, norbornene-containing polymers have high *P* values but possess low to moderate permselectivities. However, these polymers tend to undergo rearrangement within the polymer chains over time and affect the gas performance. While the studied membranes show low permeabilities with moderate permselectivities. Though the α_CO_2_/N_2__ and α_CO_2_/CH_4__ fell below the upper bound lines, these BCPs membranes are comparable to poly(IL)s and additional PNBs [24,25,30,34]. Thus, this initial proof of concept is promising and gives us an opportunity to continue work on improving the solubility of these materials and, further, develop a new class of polymers or monomers to study their structure–property relationships by tuning the side-chain substituents. For instance, the incorporation of halide groups in the pendant alkyl chains can open several opportunities as well as gas transport properties.

## 5. Conclusions

In summary, we have developed a simple and straightforward route for preparing five norbornenyl-containing imidazolium dicationic ILs [NBM-DILs] with varied connectivity and coupled with bistriflimide as a counter-anion. These [NBM-DILs] were obtained in high yields and purity was confirmed with NMR (^1^H and ^13^C) and FT-IR. Further, the [NBM-DILs] monomers were subjected to homo-, random-, and block (co) polymerization *via* ROMP. Interestingly, a series of four block copolymers (BCPs) at different feed ratios has been demonstrated with good solubility parameters, whereas insolubility was observed for the homo- and random (co) polymers. The thickness for *p*([NBM-mIm][Tf_2_N]) and BCPs membranes are in the range of 45–125 μm as prepared by the solvent casting method. All the newly developed BCPs are amorphous in nature and exhibit high molecular weight and high density with no significant difference in the intersegmental spacing. The hydrophobic components in the BCPs diluted the solubility parameter and affected the permeability of the membrane, but they enhanced CO_2_/N_2_ and CO_2_/CH_4_ permselectivities.

## Figures and Tables

**Table 1:** Details of abbreviation, structural, and molecular weight for *p*([NBM-mIm][Tf_2_N) and BCPs.

ID	Membrane	Feed Ratio ^a^ [M]:[DIL]	Density (g/cm^3^)	WAXD		GPC ^b^		
				*d*- Spacing (Å)	FWHM (Deg.)	M_n_ (Da)	M_w_ (Da)	PDI (M_n_/M_w_)
HM	*p*([NBM-mIm][Tf_2_N)	1.0:0.0	1.24 ± 0.01	4.66	12.76 ± 0.04	32,114	64,592	2.01
BCP1-C_4_	(([NBM-mIm][Tf_2_N])_1_-*b*- ([NBM-ImC_4_mIm][Tf_2_N]_2_)_0.11_)	1.0:0.11	1.25 ± 0.03	4.65	10.17 ± 0.05	95,822	182,939	1.91
BCP2-C_4_	(([NBM-mIm][Tf_2_N])_1_-*b*- ([NBM-ImC_4_mIm][Tf_2_N]_2_)_0.25_)	1.0:0.25	1.43 ± 0.07	4.75	10.74 ± 0.03	100,857	188,930	1.87
BCP1-C_6_	(([NBM-mIm][Tf_2_N])_1_-*b*- ([NBM-ImC_6_mIm][Tf_2_N]_2_)_0.11_)	1.0:0.11	1.47 ± 0.06	4.55	11.37 ± 0.05	62,820	132,728	2.11
BCP2-C_6_	(([NBM-mIm][Tf_2_N])_1_-*b*- ([NBM-ImC_6_mIm][Tf_2_N]_2_)_0.25_)	1.0:0.25	1.53 ± 0.02	4.74	10.55 ± 0.03	17,389	56,603	3.25

^a^ Same equivalent of (0.005 eq) of [G2] catalyst was used in the polymerization reactions. ^b^ Determined by GPC using poly(methyl methacrylate) standards in HFIP with 0.1% terabutylammonium triflate salts at 40 °C.

**Table 2:** Pure gas permeabilities and permselectivities of *p*([NBM-mIm][Tf_2_N]) and BCPs measured at 2 atm and 20 °C.

Membrane	Permeability ^a,b^				Permselectivity ^b^		
	*P*_CO_2__	*P*_N_2__	*P*_CH_4__	*P*_H_2__	α_CO_2_/N_2__	α_CO_2_/CH_4__	α_CO_2_/H_2__
HM	6.34 ± 0.37	0.29 ± 0.03	0.31 ± 0.02	3.98 ± 0.13	20.76 ± 0.41	20.92 ± 0.37	1.59 ± 0.05
BCP1-C_4_	9.61 ± 0.77	0.43 ± 0.03	0.55 ± 0.01	5.54 ± 0.55	19.72 ± 0.41	17.80 ± 0.76	1.69 ± 0.14
BCP2-C_4_	7.26 ± 0.15	0.23 ± 0.02	0.29 ± 0.04	4.92 ± 0.25	31.99 ± 0.63	27.61± 0.15	1.45 ± 0.07
BCP1-C_6_	11.23 ± 0.30	0.51 ± 0.01	0.58 ± 0.02	7.69 ± 0.08	21.82 ± 0.97	19.61 ± 0.39	1.48 ± 0.01
BCP2-C_6_	6.05 ± 0.29	0.21 ± 0.05	0.34 ± 0.01	3.86 ± 0.10	30.82 ± 0.37	16.28 ± 1.03	1.57 ± 0.03

^a^ Permeability are reported in barrer, where 1 barrer = 10^−10^ [cm^3^(STP) cm]/(cm^2^ s cmHg). ^b^ Measurements are performed in triplicates. The average and error values for permeabilities and permselectivities were calculated from each individual gas run.

## Data Availability

The data measured in this study are available on request form the corresponding author.

## Supplementary Materials

The following are available online at https://www.mdpi.com/article/10.3390/membranes12030264/s1, Figure S1: Synthesis of intermediates 3-(4-bromo(iodo)alkyl)-1-methyl-1H-imidazol-3-ium bromide (iodide) salts; Figure S2: ^1^H NMR spectrum for [C_4_BrmIm]Br; Figure S3: ^1^H NMR spectrum for [C_6_BrmIm]Br; Figure S4: ^1^H NMR spectrum for [C_10_BrmIm]Br; Figure S5: ^1^H NMR spectrum for [((C_2_O)_3_C_2_I)ImIm]I; Figure S6: ^1^H NMR spectrum for [NBM-Br]; Figure S7: ^1^H NMR spectrum for [NBM-Im]; Figure S8: ^1^H NMR spectrum for [NBM-ImC_4_Br]Br; Figure S9: Structures for six norbornenyl-containing imidazolium-based mono- and di-cationic ILs studied in this work; Figure S10: ^13^C NMR spectrum for [NBM-ImC_4_mIm][Tf_2_N]_2_; Figure S11: ^13^C NMR spectrum for [NBM-ImC_4_m(2-m)Im][Tf_2_N]_2_; Figure S12: ^13^C NMR spectrum for [NBM-ImC_6_mIm][Tf_2_N]_2_; Figure S13: ^13^C NMR spectrum for [NBM-ImC_10_mIm][Tf_2_N]_2_; Figure S14: ^13^C NMR spectrum for [NBM-Im((C_2_OC_2_)_2_O)mIm][Tf_2_N]_2_; Figure S15: FT-IR spectrum for NBM-containing imidazolium-based ILs; Figure S16: ^1^H NMR spectrum for *p*([NBM-ImC_6_mIm][Tf_2_N]_2_); Figure S17: ^1^H NMR spectrum for *p*([NBM-ImC_4_m(2-m)Im][Tf_2_N]_2_); Table S1: Optimizing the reaction conditions for effective polymerization of [NBM-DILs][Tf_2_N]_2_; Figure S18: ^1^H NMR spectrum for BCP1-C_6_; Figure S19: ^1^H NMR spectrum for BCP2-C_6_; Figure S20: FT-IR spectra for BCPs; Figure S21: Flexibility of membranes; Table S2: Pure gas diffusivity and solubility coefficients.
