# Supplementary material for: Poly(ionic liquid)s with Dicationic Pendants as Gas Separation Membranes
Source: Membranes (Basel). 2022 Feb 25;12(3):264. doi: 10.3390/membranes12030264 (PMC8954690; doi:10.3390/membranes12030264)
Supplement: Supplementary file 1 [file membranes-12-00264-s001.zip › membranes-1589811.pdf]

membranes

Supplementary Materials:

Poly(Ionic Liquid)s with Dicationic Pendants as Gas Separation Membranes

Sudhir Ravula [1], Kathryn E. O'Harra [1], Keith A. Watson [2] and Jason E. Bara [1,*]

[1] Department of Chemical & Biological Engineering, University of Alabama, Tuscaloosa, AL 35487, USA; sravula@ua.edu (S.R.); keoharra@ua.edu (K.E.O.)
[2] Department of Chemistry & Biochemistry, University of Alabama, Tuscaloosa, AL 35487, USA; kawatson3@crimson.ua.edu
* Correspondence: jbara@eng.ua.edu (J.E.B)

The supporting data referenced in the manuscript: "Poly(Ionic Liquid)s with Dicationic Pendants as Gas Separation Membranes". Structures, NMR (^{1}H and ^{13}C), optimization conditions and gas separation properties (diffusivity and solubility) results are shown in the following sections.

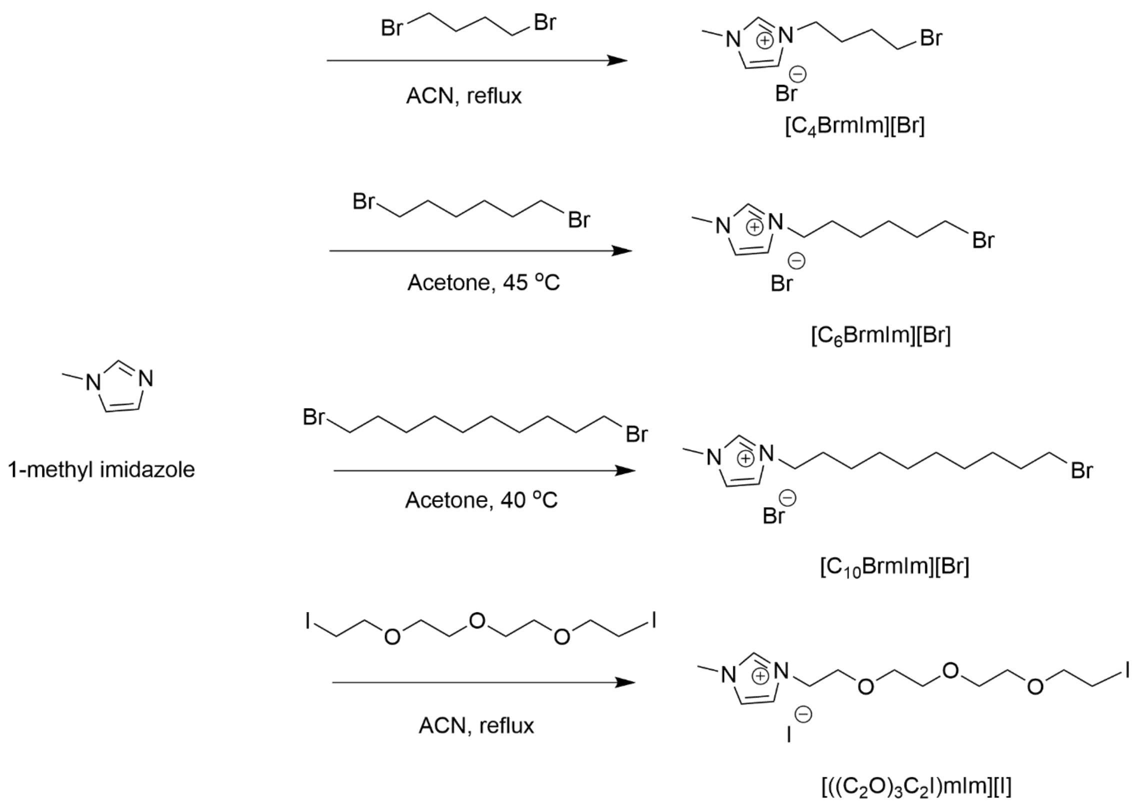

Figure S1. Synthesis of intermediates 3-(4-bromo(iodo)alkyl)-1-methyl-1*H*-imidazol-3-ium bromide (iodide) salts and their corresponding ^{1}H NMR spectra were shown below.

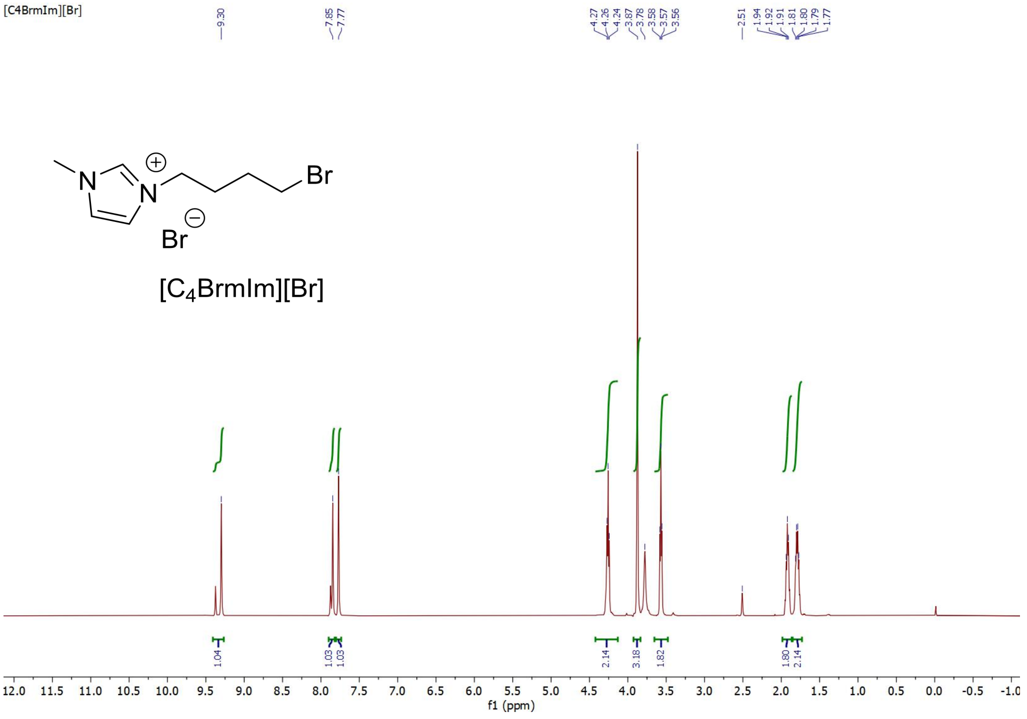

Figure S2. ^{1}H NMR spectrum for $[C_4BrmIm]Br$.

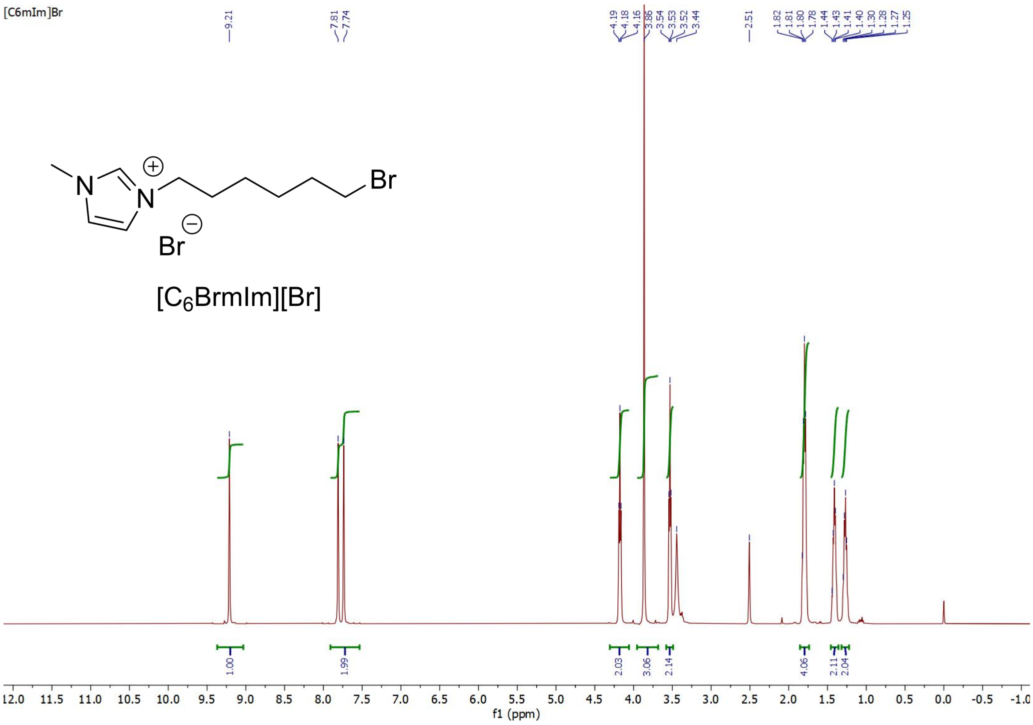

Figure S3. ^{1}H NMR spectrum for [C_6BrmIm]Br.

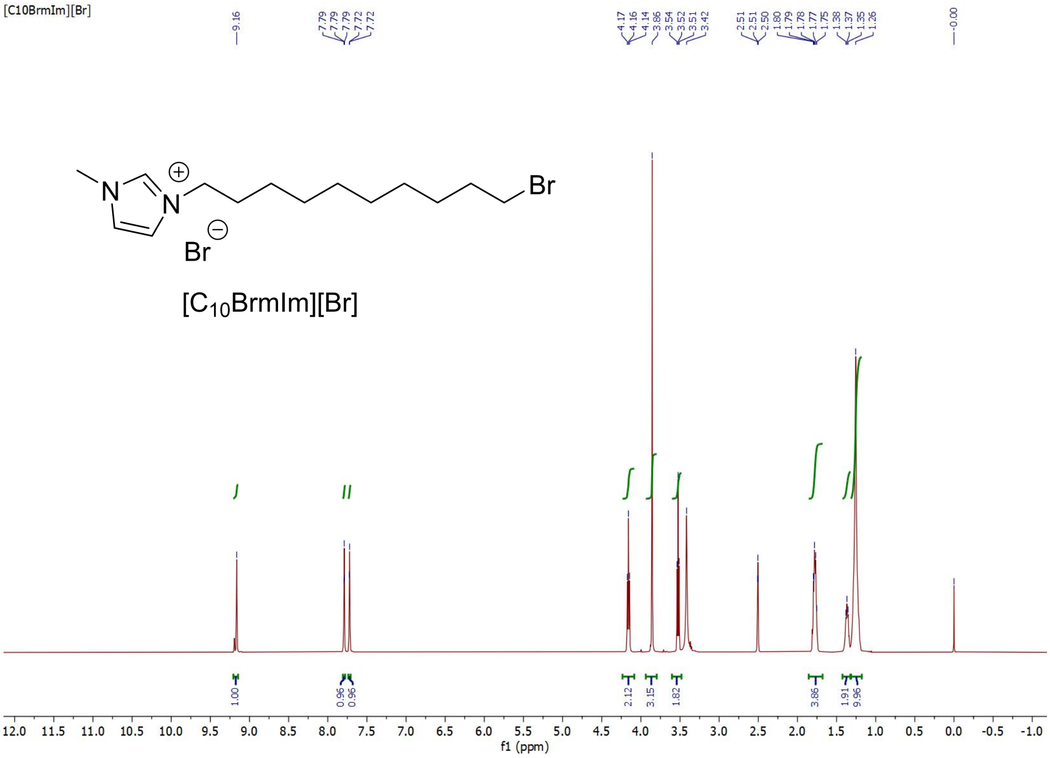

Figure S4. ^{1}H NMR spectrum for [C_{10}BrmIm]Br.

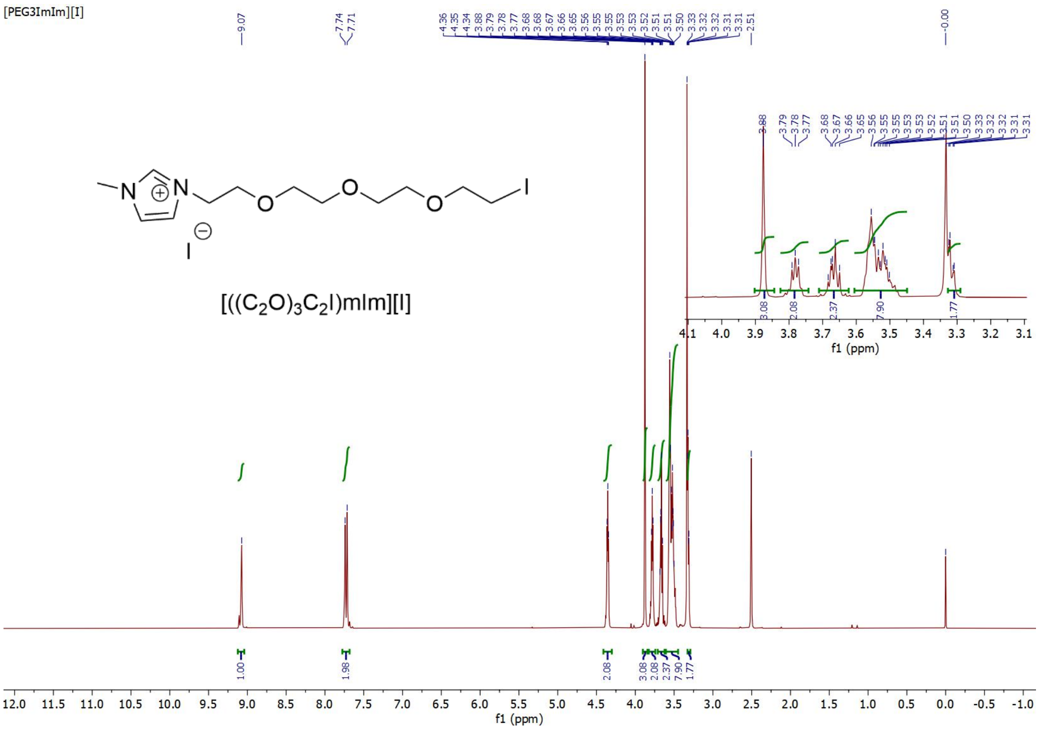

Figure S5. ^{1}H NMR spectrum for [((C_2O)$_3$$C_2$I)mIm]I. Inset image shows the proton region from 3.1 to 4.1 ppm.

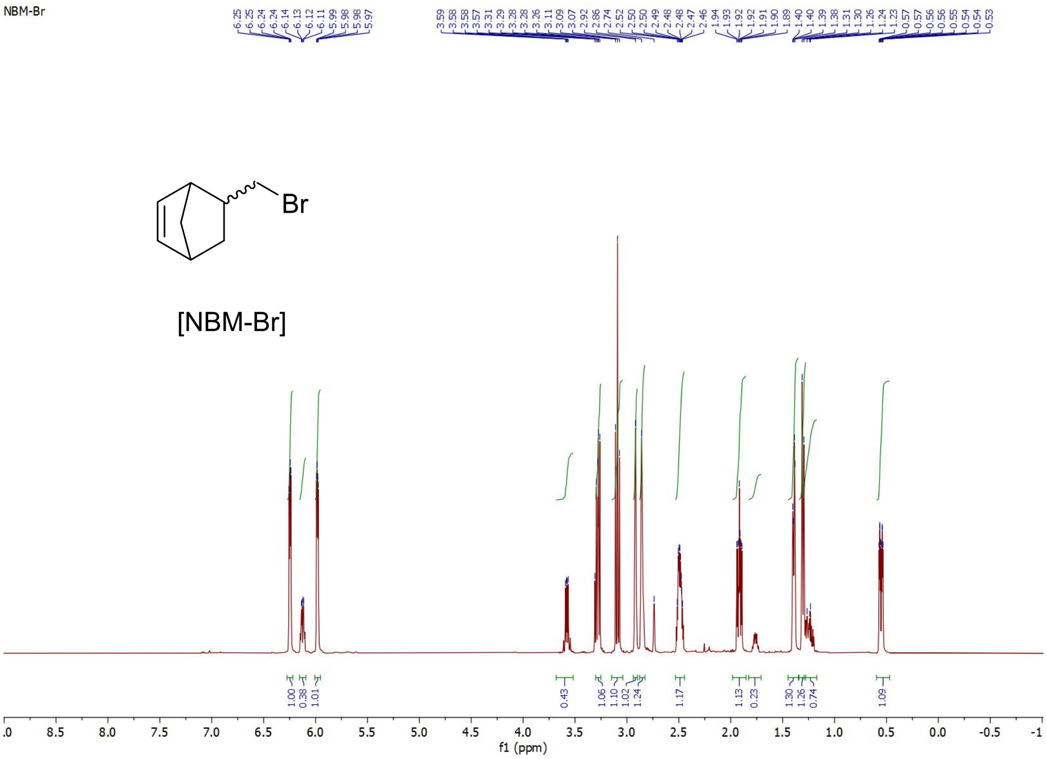

Figure S6. ^{1}H NMR spectrum for [NBM-Br].

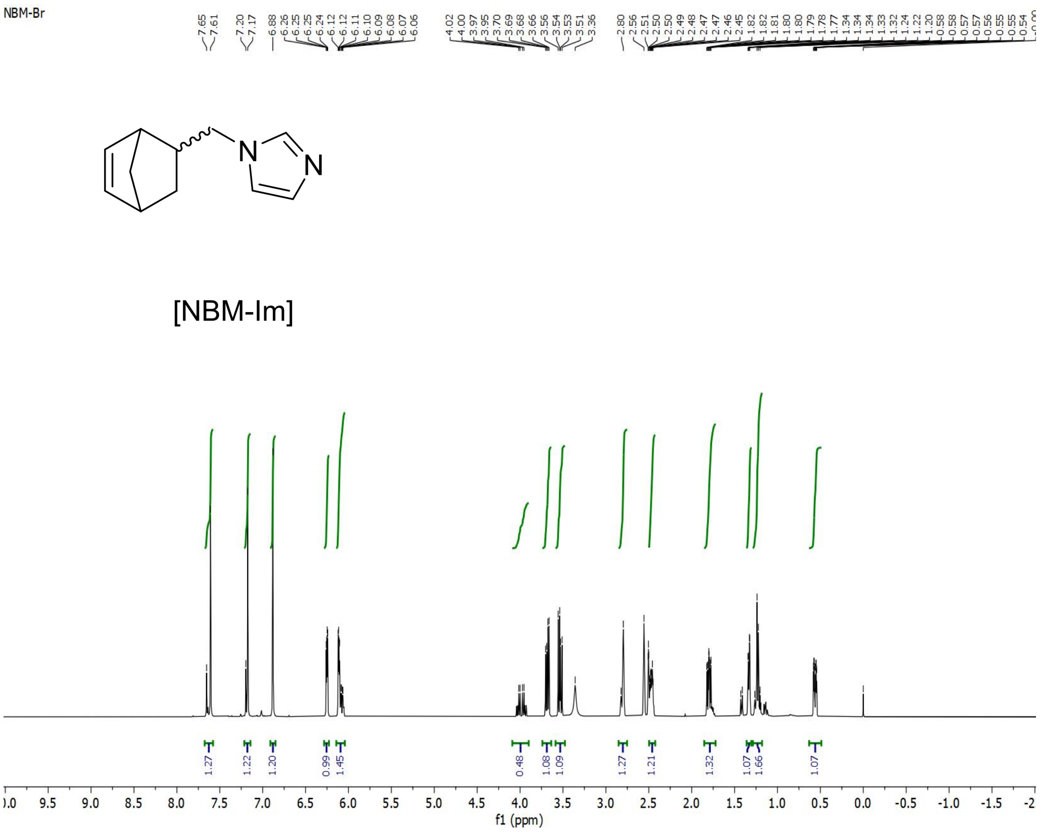

Figure S7. ^{1}H NMR spectrum for [NBM-Im].

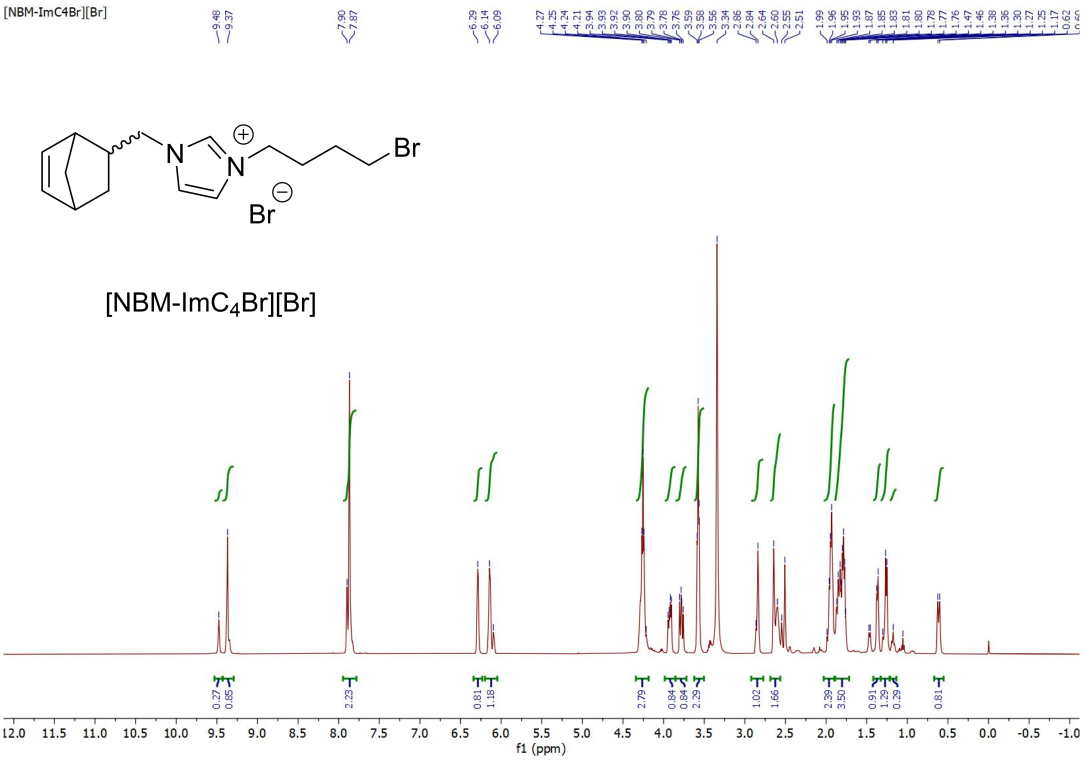

Figure S8. ^{1}H NMR spectrum for [NBM-ImC$_4$Br] Br.

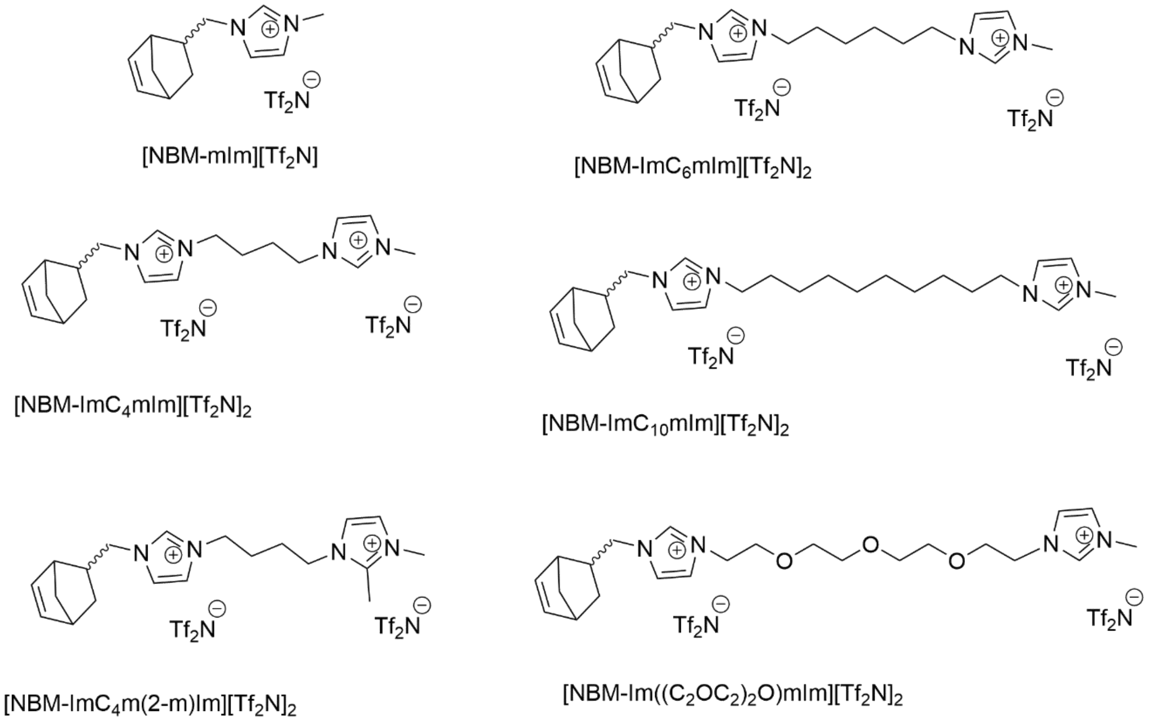

Figure S9. Structures for six norbornenyl-containing imidazolium-based mono- and dicationic ILs studied in this work.

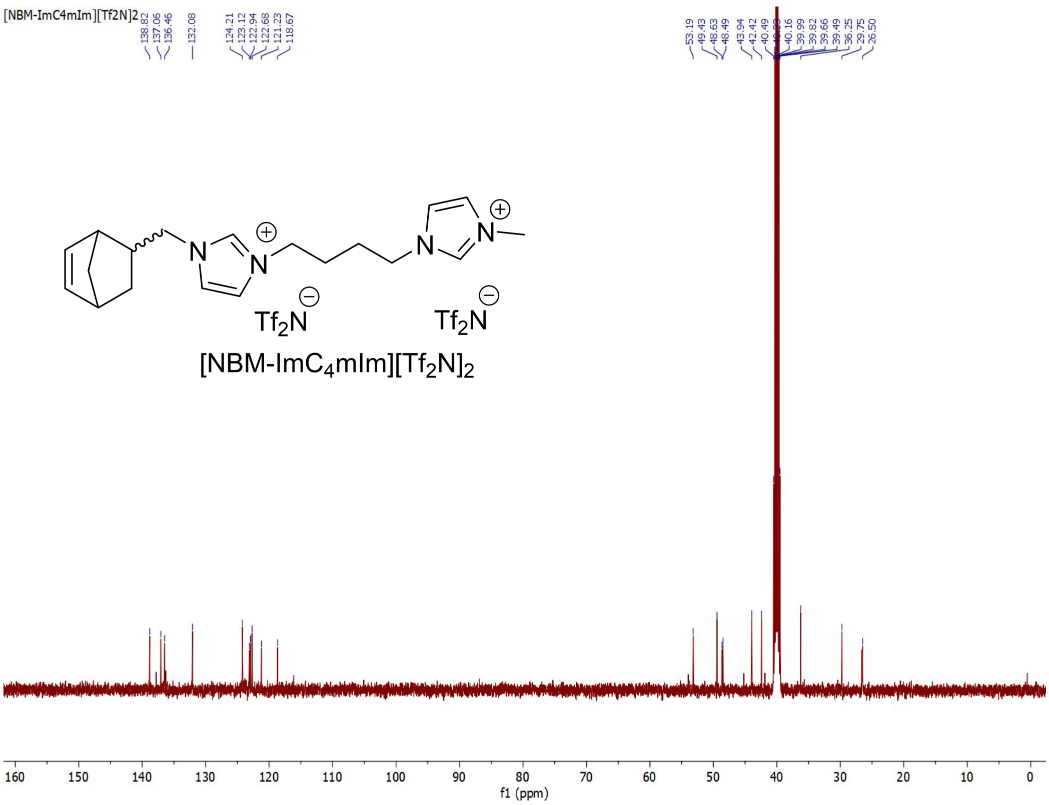

Figure S10. ^{13}C NMR spectrum for [NBM-ImC$_4$mIm][Tf$_2$N]$_2$.

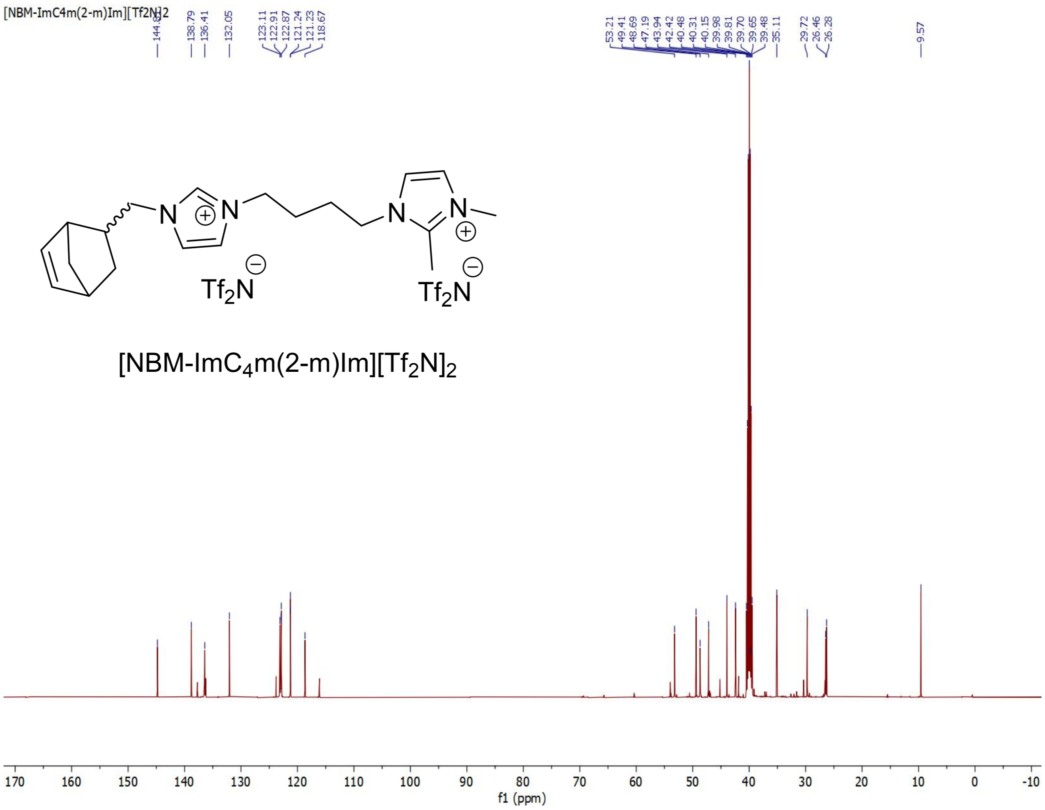

Figure S11. ^{13}C NMR spectrum for [NBM-ImC$_4$m(2-m)Im][Tf$_2$N]$_2$.

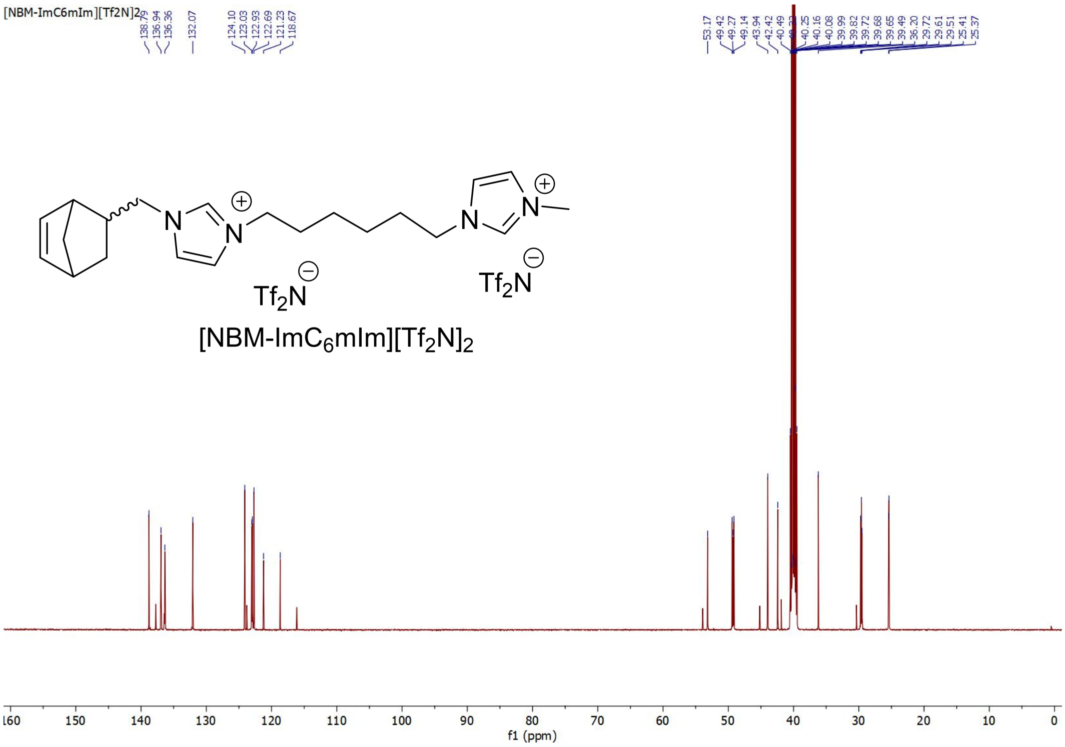

Figure S12. ^{13}C NMR spectrum for [NBM-ImC$_6$mIm][Tf$_2$N]$_2$.

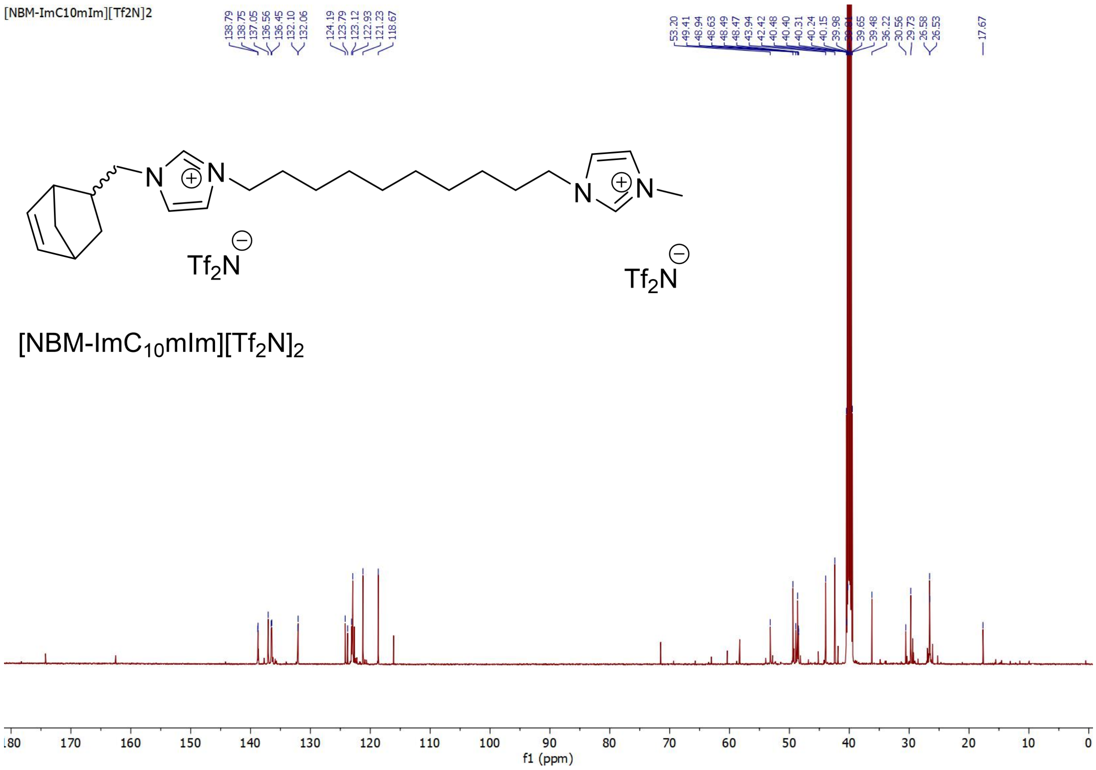

Figure S13. ^{13}C NMR spectrum for [NBM-ImC$_{10}$mIm][Tf$_2$N]$_2$

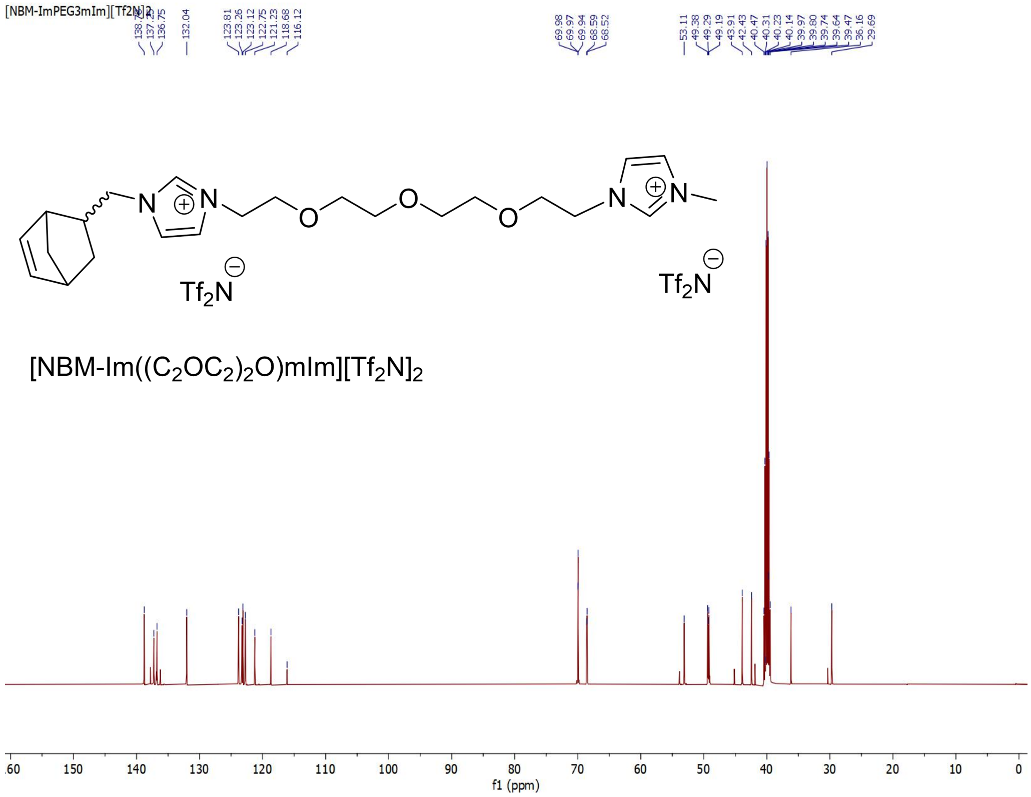

Figure S14. ^{13}C NMR spectrum for [NBM-Im((C$_2$OC$_2$)$_2$O)mIm][Tf$_2$N]$_2$.

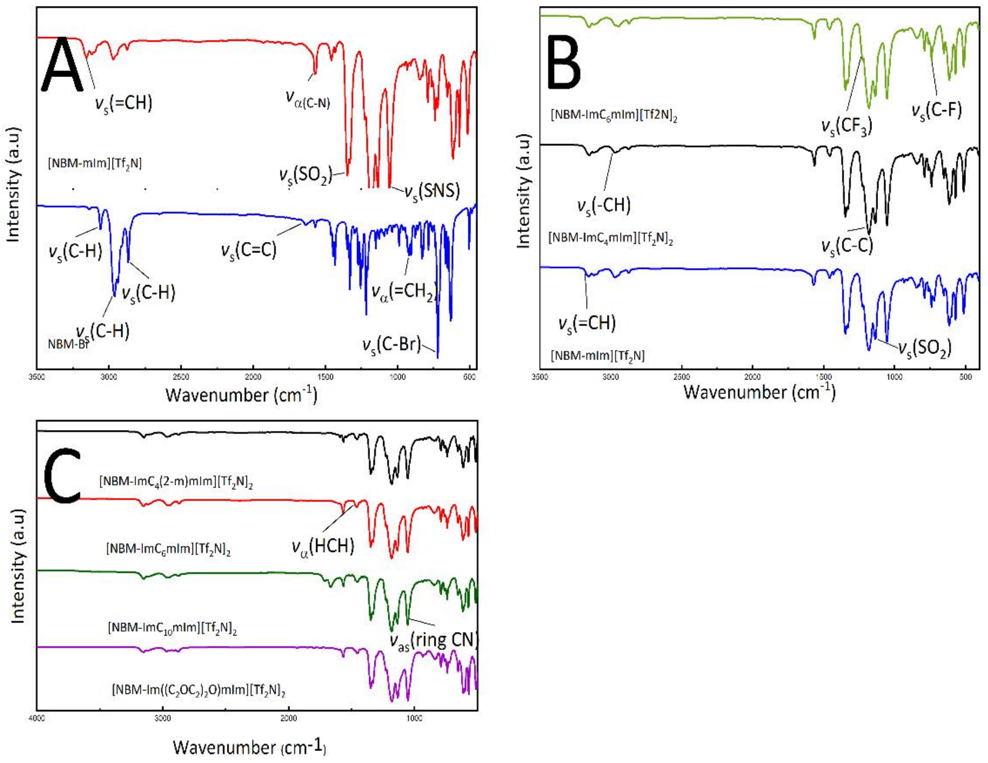

Figure S15. FT-IR spectrum for NBM-containing imidazolium-based ILs. (**A**) FT-IR spectrum for NBM-Br and [NBM-mIm][Tf_2N] (**B**) FT-IR spectrum for [NBM-mIm][Tf_2N], [NBM-ImC_4mIm][Tf_2N], and [NBM-ImC_6mIm][Tf_2N] (**C**) FT-IR spectrum for imidazolium-based dicationic IL monomers.

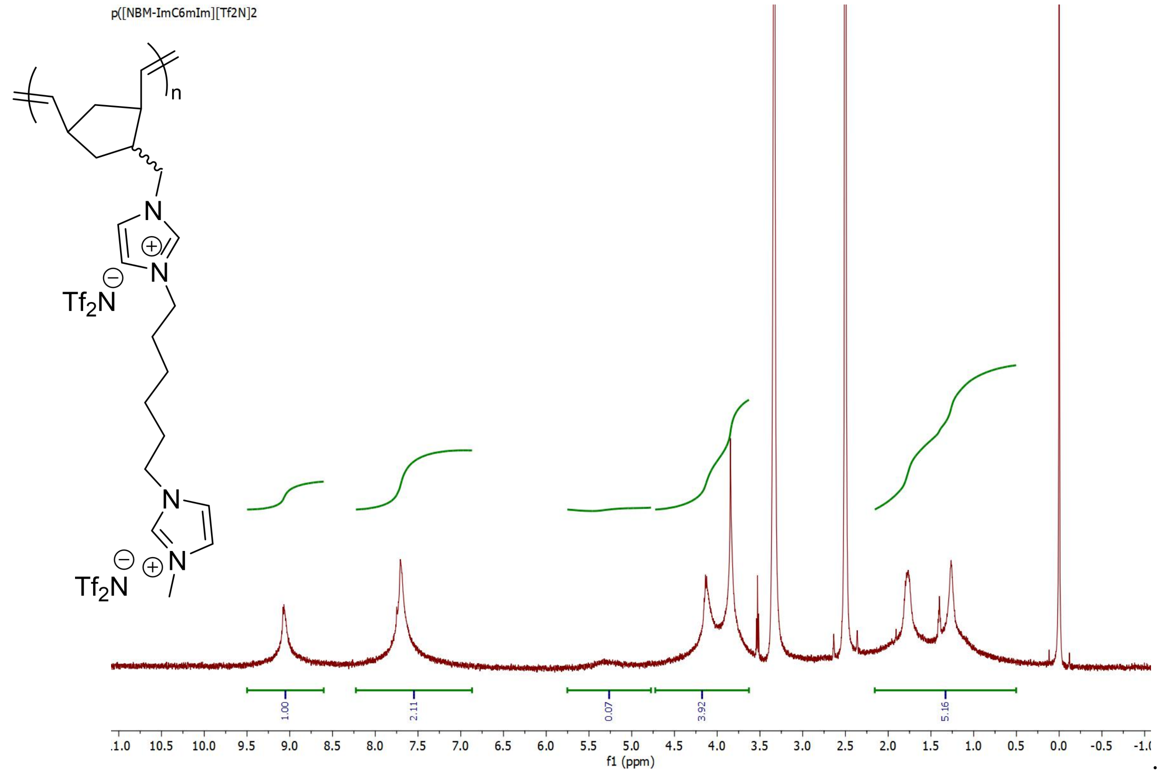

Figure S16. ^{1}H NMR spectrum for *p*([NBM-ImC$_6$mIm][Tf$_2$N]$_2$). Sparingly soluble in DMSO-d_6.

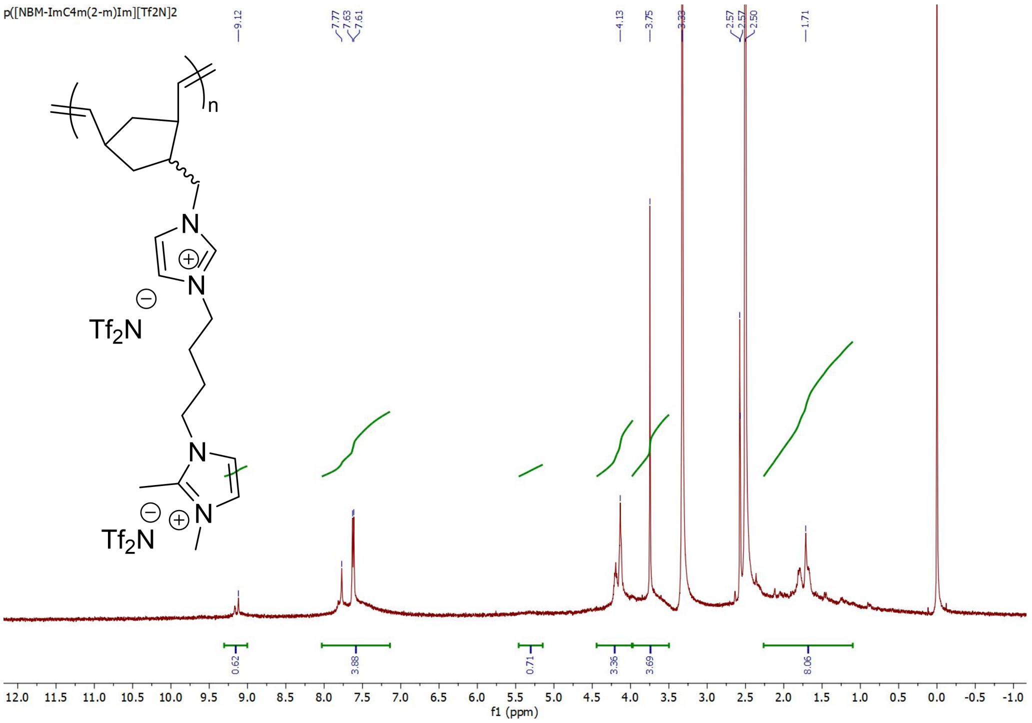

Figure S17. ^{1}H NMR spectrum for *p*([NBM-ImC$_4$m(2-m)Im][Tf$_2$N]$_2$). Sparingly soluble in DMSO-d_6.

Table S1. Optimizing the reaction conditions for effective polymerization of [NBM-DILs][Tf_2N]$_2$.

Entry	Monomer	Feed ratio [M]:[G2]	Time	Temp. (°C)	Solvent	Polymer Solubility
1	[NBM-mIm][Tf_2N]	200:1	4 h	RT	DCM	Soluble
				RT	DCM	Insoluble
2	[NBM-ImC_4mIm][Tf_2N]		30 min-24 h	20–100	DMF	Insoluble
		200:1 to 1000:1		20–100	DMF/DCM (2:1)	Insoluble
				20–70	THF	Insoluble
3	[NBM-ImC_6mIm][Tf_2N]$_2$		30 min-24 h	RT	DCM	Insoluble
4	[NBM-ImC_{10}mIm][Tf_2N]$_2$		30 min-24 h	RT	DCM	Insoluble
5	[NBM-Im((C_2OC_2)$_2$O)mIm][Tf_2N]$_2$		30 min-24 h	RT	DCM	Insoluble

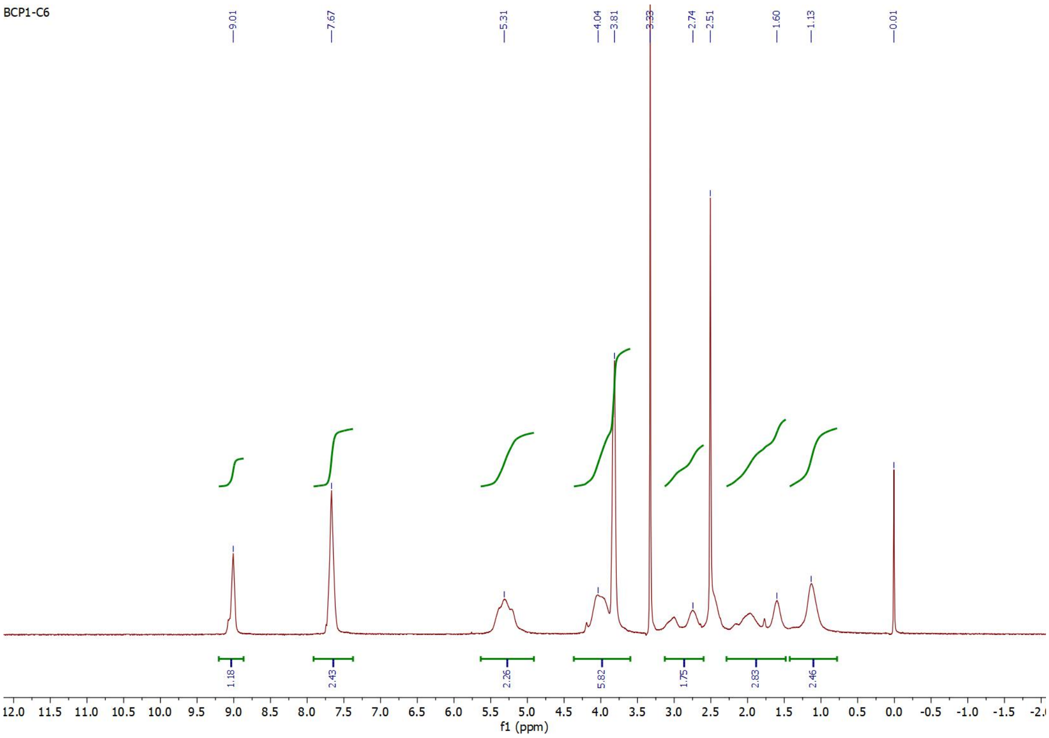

Figure S18. ^{1}H NMR spectrum for BCP1-C_6.

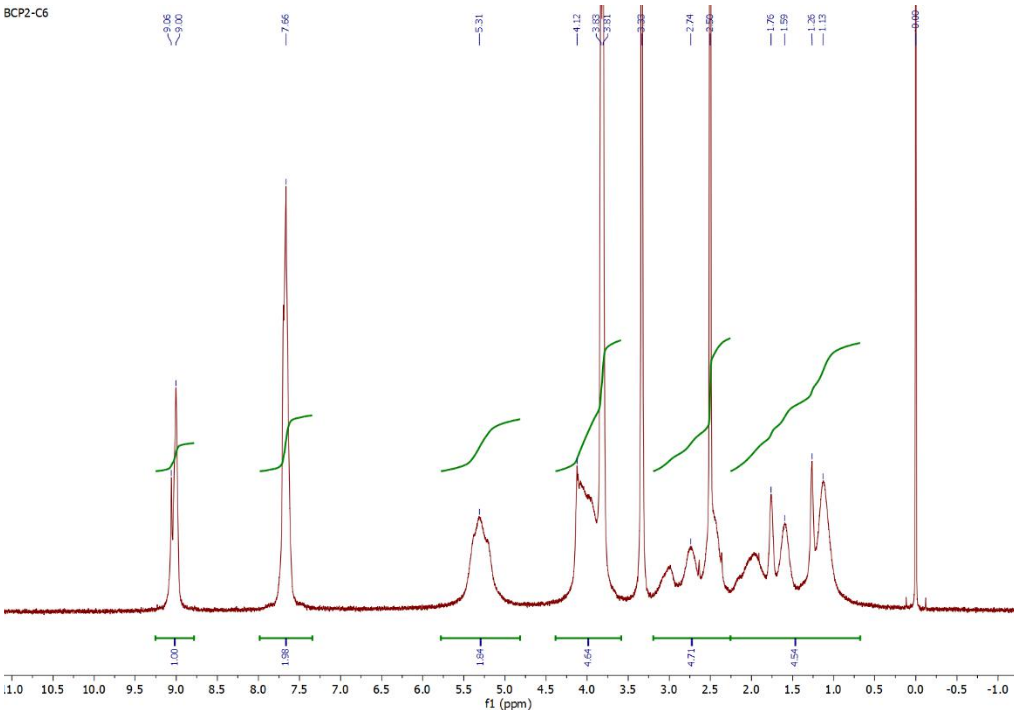

Figure S19. ^{1}H NMR spectrum for BCP2-C_6.

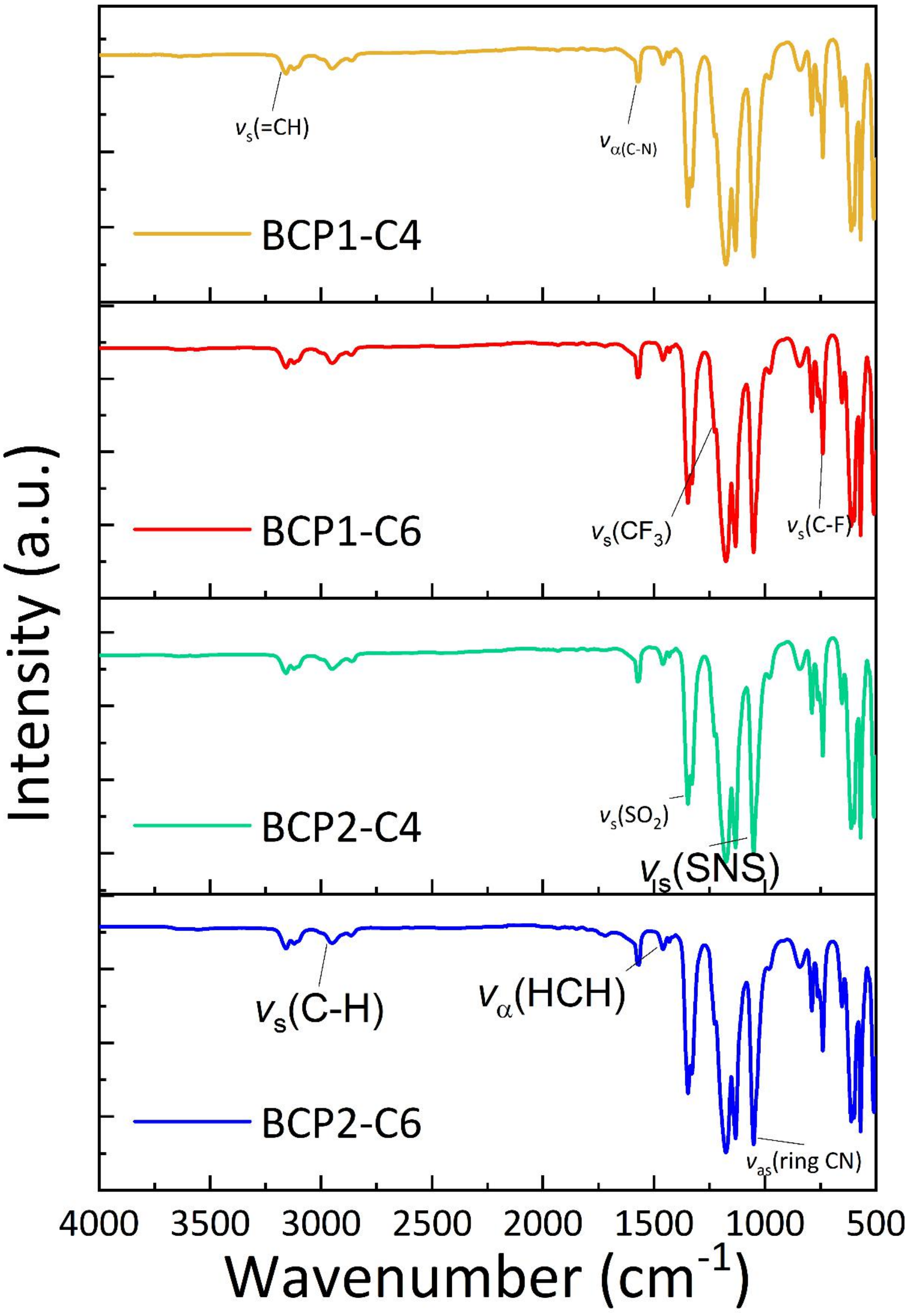

Figure S20. FT-IR data for BCPs.

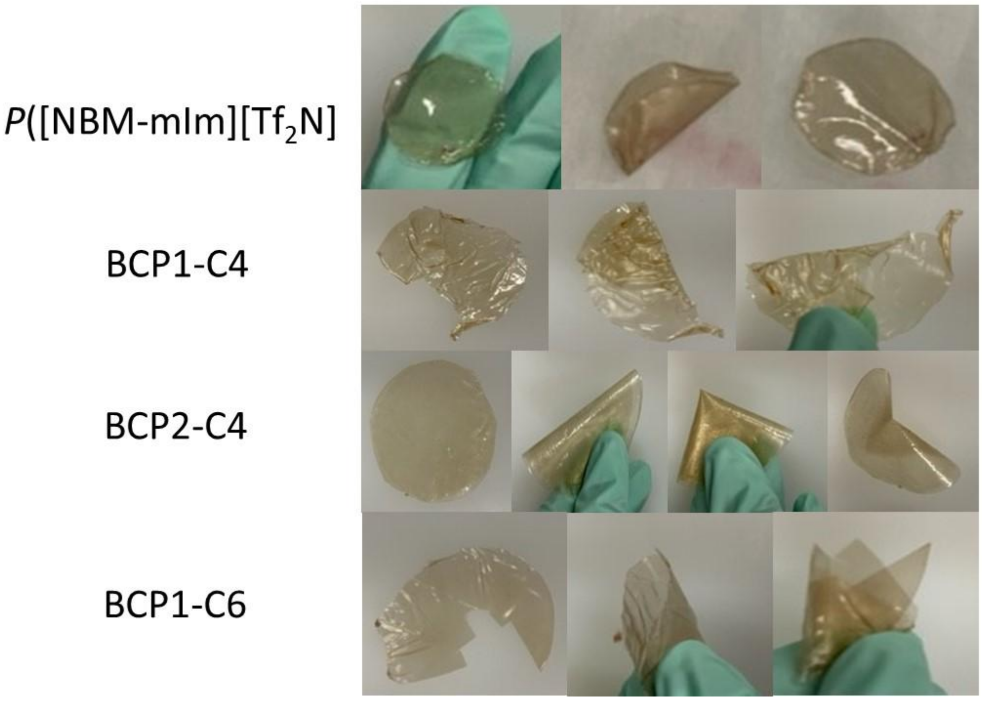

Figure S21. Flexibility of membranes.

Table S2. Pure gas diffusivity and solubility coefficients.

ID	**Diffusivity**[a,b]				**Solubility**[a,c]			
	D_{CO2}	D_{N2}	D_{CH4}	D_{H2}	S_{CO2}	S_{N2}	S_{CH4}	S_{H2}
HM	1.62 ± 0.10	0.39 ± 0.08	0.77 ± 0.39	NA	40.3 ± 4.77	6.88 ± 1.69	2.37 ± 1.08	NA
BCP1-C_4	25.37 ± 5.7	2.42 ± 0.40	2.84 ± 0.36	15.37 ± 2.51	4.08 ± 0.27	2.09 ± 0.62	2.10 ± 0.03	3.76 ± 0.50
BCP2-C_4	8.35 ± 0.98	NA	6.71 ± 1.99	13.97 ± 0.46	9.23 ± 0.01	NA	0.42 ± 0.01	3.55 ± 0.38
BCP1-C_6	12.96 ± 2.47	4.08 ± 1.66	5.06 ± 0.84	28.21 ± 5.57	8.84 ± 1.42	1.02 ± 0.74	1.16 ± 0.15	2.05 ± 0.63
BCP2-C_6	10.40 ± 2.46	1.45 ± 0.42	1.41 ± 0.37	9.81 ± 2.64	5.73 ± 0.94	1.33 ± 0.89	2.08 ± 0.85	3.64 ± 0.43

The diffusivity and solubility coefficients were measured at 2 atm and 20 °C. [a]Measurements are performed in triplicates. [b]Diffusivity coefficient (10^{-9} cm^2/s). [c]Solubility coefficient (10^{-2} $cm^3_{(STP)}$ cm^{-3} $cmHg^{-1}$).
